# A functional mini-GDE transgene corrects impairment in models of glycogen storage disease type III

**DOI:** 10.1172/JCI172018

**Published:** 2024-01-16

**Authors:** Antoine Gardin, Jérémy Rouillon, Valle Montalvo-Romeral, Lucille Rossiaud, Patrice Vidal, Romain Launay, Mallaury Vie, Youssef Krimi Benchekroun, Jérémie Cosette, Bérangère Bertin, Tiziana La Bella, Guillaume Dubreuil, Justine Nozi, Louisa Jauze, Romain Fragnoud, Nathalie Daniele, Laetitia Van Wittenberghe, Jérémy Esque, Isabelle André, Xavier Nissan, Lucile Hoch, Giuseppe Ronzitti

**Affiliations:** 1 Genethon, Evry, France.; 2 Université Paris-Saclay, Univ Evry, Inserm, Genethon, Integrare research unit UMR_S951, Evry, France.; 3 CECS, I-STEM, Institute for Stem Cell Therapy and Exploration of Monogenic Diseases, Corbeil-Essonnes, France.; 4 Toulouse Biotechnology Institute, TBI, Université de Toulouse, CNRS, INRAE, INSA, Toulouse, France.

**Keywords:** Metabolism, Therapeutics, Carbohydrate metabolism, Gene therapy, Neuromuscular disease

## Abstract

Glycogen storage disease type III (GSDIII) is a rare inborn error of metabolism affecting liver, skeletal muscle, and heart due to mutations of the *AGL* gene encoding for the glycogen debranching enzyme (GDE). No curative treatment exists for GSDIII. The 4.6 kb GDE cDNA represents the major technical challenge toward the development of a single recombinant adeno-associated virus–derived (rAAV-derived) vector gene therapy strategy. Using information on GDE structure and molecular modeling, we generated multiple truncated GDEs. Among them, an N-terminal–truncated mutant, ΔNter2-GDE, had a similar efficacy in vivo compared with the full-size enzyme. A rAAV vector expressing ΔNter2-GDE allowed significant glycogen reduction in heart and muscle of *Agl*^–/–^ mice 3 months after i.v. injection, as well as normalization of histology features and restoration of muscle strength. Similarly, glycogen accumulation and histological features were corrected in a recently generated *Agl*^–/–^ rat model. Finally, transduction with rAAV vectors encoding ΔNter2-GDE corrected glycogen accumulation in an in vitro human skeletal muscle cellular model of GSDIII. In conclusion, our results demonstrated the ability of a single rAAV vector expressing a functional mini-GDE transgene to correct the muscle and heart phenotype in multiple models of GSDIII, supporting its clinical translation to patients with GSDIII.

## Abstract 2

Single adeno-associated virus vector gene therapy corrects muscle and heart impairment in rodent and human models of glycogen storage disease type III.

## Graphical abstract

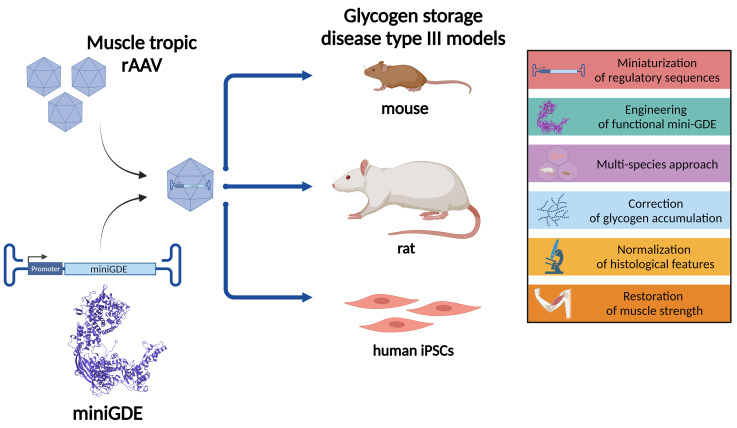

## Introduction

Glycogen storage disease type III (GSDIII) is a rare inborn error of metabolism, with an incidence of 1 in 100,000, caused by mutations in the *AGL* gene, which encodes for the glycogen debranching enzyme (GDE; also known as amylo-α-1,6-glucosidase) (1, 2). GDE acts along with glycogen phosphorylase to degrade glycogen in the cytosol of virtually any cell. In liver and muscle, GDE function is required to maintain glycemia and to sustain fast muscle contraction, respectively (1).

The earliest manifestations of GSDIII, such as fasting hypoglycemia, hepatomegaly, elevation of liver enzymes, and growth retardation, usually appear from early childhood (1, 2). A severe myopathy develops during adolescence, with exercise intolerance and muscle weakness followed by loss of ambulation in adult patients (2–4). Histological analysis of muscle biopsies from patients with GSDIII shows glycogen accumulation in large vacuoles, which disrupts the myofibril architecture (5). Most adult patients also display left ventricle hypertrophy on echocardiography, but only 15% have overt cardiomyopathy (2, 4). Although it was long thought that liver involvement improved with age, recent studies report liver fibrosis from an early age with cirrhosis and liver tumor development in adult patients (3, 6). To date, no curative treatment is available for patients with GSDIII, and therapeutic options mostly consist of frequent meals, the use of complex carbohydrates such as uncooked cornstarch, and a high-fat high-protein diet (1, 7). Dietary management reduces the frequency of hypoglycemia and improves, in some cases, the cardiac phenotype, but has little effect on the myopathy (1, 7).

Gene therapy using recombinant adeno-associated virus (rAAV) vectors is a promising strategy to treat inherited diseases (8, 9) and has already been successfully used in spinal muscular atrophy (10), hemophilia A and B (11, 12), and congenital blindness (13), with marketing approval in the treatment of these conditions. Multiple clinical trials are currently ongoing for liver or muscle genetic diseases, with encouraging results (9). One of the major constraints in the use of rAAV for gene transfer is that its encapsidation size is limited to 5 kb, including the inverted terminal repeats (ITRs) (8). In the setting of GSDIII, the 4.6 kb GDE cDNA represents a technical challenge toward the development of a single vector strategy for GSDIII (14). Dual AAV vector strategies relying on the presence of homologous recombination sequences or on inteins peptides in each vector to achieve the expression of the full length protein, e.g the full-length GDE, in the cell (15–17). A few years ago, our team published a proof-of-concept of GSDIII correction using a dual overlapping rAAV gene therapy strategy in which 2 vectors were coinjected, each encoding half of the expression cassette (15). By using 2 distinct dual rAAV vector approaches, we demonstrated complete correction of the muscle and heart phenotype, but only partial correction of the liver disease (15). An improvement of this approach, based on the use of a tandem liver-muscle promoter (18) in combination with an immunosuppressive treatment, was recently reported (19). Despite ongoing optimization (16, 17), the dual vector strategy has several limitations: it requires 2 vectors and a consequent 2-times-higher dose, the recombination efficacy could be suboptimal, and the expression of truncated proteins derived from nonrecombined genomes could trigger potential immune toxicities. Another strategy has been developed using a bacterial debranching enzyme (pullulanase), whose 2.2 kb cDNA is short enough to be encapsidated in a single rAAV vector (20). Although this approach allows initial liver and muscle correction (20), it led to immune response toward the bacterial protein and subsequent loss of correction (21). Given the limitations of the existing rAAV gene transfer technology, a strategy based on a single vector expressing a shorter and active form of GDE may represent a valid solution for a safe and efficient rAAV gene therapy for GSDIII in people.

In patients with GSDIII, the liver displays liver fibrosis from early childhood (2, 3, 6) and severe, sometimes lethal, liver toxicities have been reported in clinical trials using high-dose rAAVs (22, 23). Based on these considerations, we focused on the treatment of heart and muscle impairment while detargeting the liver with a recently developed muscle-tropic rAAV vector (24).

Here, using *Agl*-KO mouse and rat models, as well as a human cellular model of GSDIII, we demonstrate the possibility of engineering a single vector encoding a mini-GDE enzyme to correct the muscular and cardiac manifestations of the disease, the major disease burden in adults with GSDIII (2, 3, 7).

## Results

### Generation of a single-vector approach for GSDIII based on a functional, truncated GDE.

Our team previously demonstrated the possibility to correct the muscle and heart phenotype of a GSDIII mouse model (*Agl*^–/–^ mice) using a dual vector approach harboring the cytomegalovirus (CMV) promoter (15). A GDE expression cassette containing the human full-length GDE cDNA and the CMV promoter used in the previous study is close to 6 kb, largely beyond the packaging size limitation of a single rAAV vector. As a first step toward the optimization of the gene therapy approach for GSDIII, we carefully optimized each component of the transgene expression cassette to reduce their size at minimum.

First, we compared in vivo the strength of promoters with proven expression in muscle and sizes spanning 334 to 1,050 bp such as miniCMV, SPc5-12, tMCK, eSyn, Desmin, or CK6 (25, 26) (Supplemental Figure 1A; supplemental material available online with this article; https://doi.org/10.1172/JCI172018DS1). Mice were i.v. injected with rAAV9 encoding the reporter gene mouse secreted embryonic alkaline phosphatase (mSEAP) under the control of each promoter, and mSEAP expression was assessed in 2 skeletal muscles. MiniCMV, one of the shortest promoters (351 bp), showed the highest mSEAP expression with similar vector genome copy numbers (VGCN) among all tested promoters (Supplemental Figure 1, B and C) in triceps and quadriceps.

Muscle targeting using rAAV usually requires high doses and may expose patients to liver overload and potential toxicities (22, 23). Our team has already reported the generation of a potent muscle-targeting and liver-detargeting rAAV chimeric capsid referred to as rAAV-MT (24). A biodistribution study performed a month after i.v. injection in mice confirmed that rAAV-MT enabled higher expression of the reporter gene in skeletal muscles as well as a pronounced reduction of expression in liver (Supplemental Figure 2, A and B). Introns are known to increase the stability of mRNA, the export to the nucleus, and, ultimately, enhance protein production (27). To understand the requirement of the intron in our expression cassette, we assessed the ability of 2 similar, oversized expression cassettes with or without the SV40 intron. For this and the following experiments to comparatively evaluate the efficacy of the truncated enzymes, intramuscular injections were used (Supplemental Figure 3A). Intramuscular vector administration in *Agl*^–/–^ mice represents a fast method to evaluate the activity of GDE toward its native substrate in a diseased context, in the absence of a reliable and robust in vitro test to evaluate enzymatic activity. Following intramuscular injection, similar vector transduction and GDE expression, as measured by VGCN and Western blot, resulted in a significant decrease of glycogen accumulation in the injected muscle of mice treated with both vectors (Supplemental Figure 3, B–E), suggesting that the presence of an intron is not necessary to achieve a high level of GDE expression.

Without the intron, the size of the expression cassette enables the generation of oversized rAAV vectors to assess efficacy in our *Agl^–/–^* mouse model after i.v. injection. We then tested 2 single rAAV vectors, containing either a poly adenylation (poly A) signal of 58 base pairs (pA58) or of 169 (bGh) base pairs, encapsidated in rAAV-MT (Supplemental Figure 4A). Three months after injection, muscle transduction and GDE expression, respectively measured by VGCN and Western blot, were similar in all evaluated muscles, including heart (Supplemental Figure 4, B–D). The low levels of VGCN observed in muscle likely reflected the use of an oversized AAV vector cassette with suboptimal packaging. Similar but partial efficacy was observed with the 2 expression cassettes, with 50% to 60% reduction of glycogen measured in heart, triceps, quadriceps, soleus, and extensor digitorum longus (EDL) muscles (Supplemental Figure 5A and Supplemental Table 1). In line with partial reduction of tissue glycogen, histological analysis revealed a limited normalization of the muscle histology (Supplemental Figure 5B).

Taken together, these results allowed us to select the minimal regulatory sequences necessary to efficiently express GDE with a single vector. However, the optimized transgene expression cassette including the native GDE cDNA had a size of 5.3 kb and showed partial efficacy in *Agl^–/–^* mice, thus supporting efforts to reduce the size of the GDE transgene.

**Figure 1:**
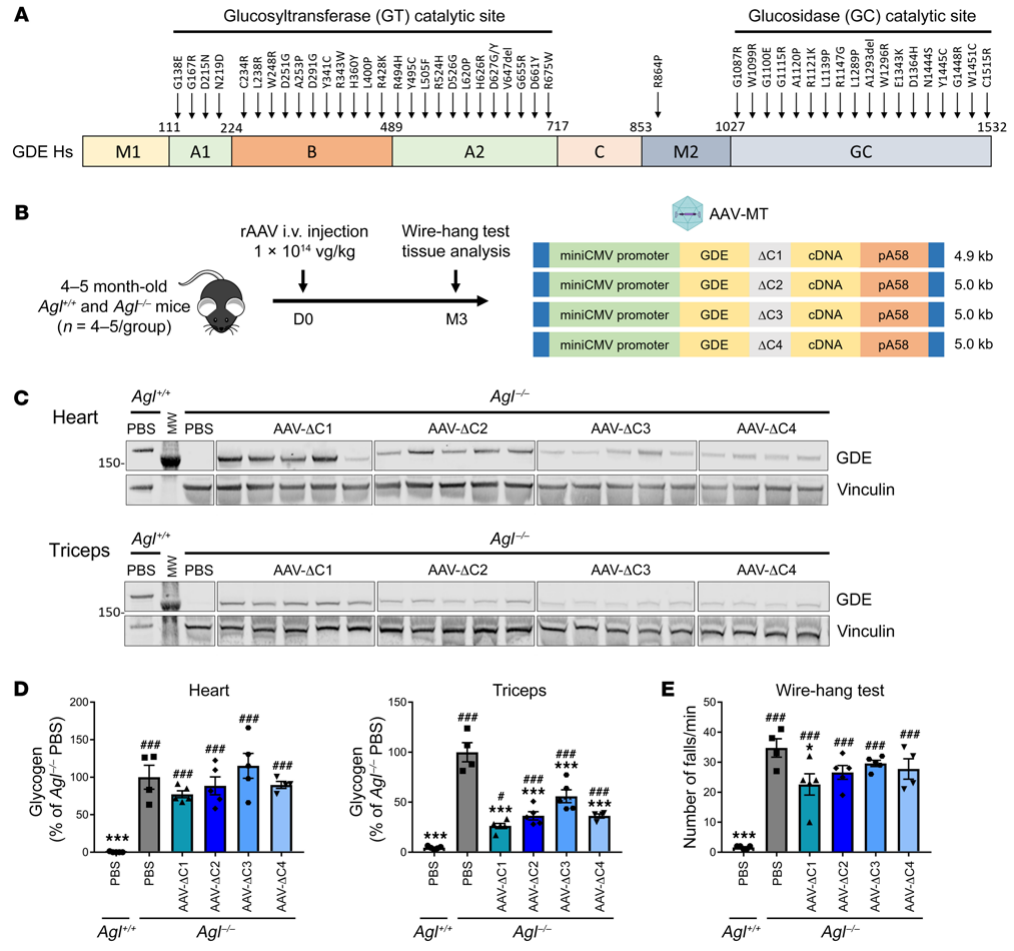
Generation of truncated GDE polypeptides in the C domain. (**A**) Schematic representation of the human GDE cDNA, depicting the different functional domains and the 2 catalytic sites (28), as well as the position of missense mutations reported in patients with GSDIII (2, 5, 6, 28, 31–38). (**B**) 4–5 month-old male *Agl*^–/–^ mice were injected in the tail vein with an rAAV-MT vector encoding ΔC1 to ΔC4 GDE mutants, at the dose of 1 × 10^14^ vg/kg. PBS-injected *Agl*^+/+^ or *Agl*^–/–^ mice were used as controls. (**C**) Western blot analysis of GDE and vinculin expression in heart and triceps 3 months after vector injection. (**D**) Glycogen content measured in heart and triceps 3 months after vector injection. (**E**) Wire-hang test expressed as the number of falls per minute, performed 3 months after vector injection. Statistical analyses were performed by 1-way ANOVA. *P < 0.05, **P < 0.01, ***P < 0.001 versus PBS-injected *Agl*^–/–^ mice; ^#^*P* < 0.05, ^##^*P* < 0.01, ^###^*P* < 0.001 versus PBS-injected *Agl*^+/+^ mice; *n* = 4–5 mice per group. All data are shown as mean ±SEM.

Although no three-dimensional structure of human GDE has been experimentally determined so far, the structure of a GDE homolog from *Candida glabrata* (PDB id: 5D06 and 5D0F), showing 38% of sequence identity with the human GDE, was solved using X-ray diffraction (28). With the emergence of AlphaFold2, an artificial intelligence system developed by DeepMind (29), high quality models are now available, notably via the AlphaFold2 database (30), where a model of human GDE can be found (entry AF-P35573-F1, Supplemental Figure 6, A and B). This model was retrieved from the database and compared with the crystallographic structure of *C*. *glabrata* GDE, enabling the identification in human GDE of the different domains described earlier for *C*. *glabrata* (28), namely M1, GT (corresponding to the assembly of 3 subdomains: A, B and C), M2, and GC (Supplemental Figure 6, C). The mapping of the missense mutations described in GSDIII patient onto the 3D model revealed the presence of most mutations in the catalytic GT and GC domains (2, 5, 6, 28, 31–38), while almost no mutations were reported in the M1 and C domains (Figure 1A). Interestingly, the crystallographic structure of inactive *C*. *glabrata* GDE in complex with maltooligosaccharides did not reveal the presence of any sugar binding site in these 2 domains (adapted from PDB id: 5D06 and 5D0F, Supplemental Figure 6B). These considerations support the assumption that these 2 domains may not play a relevant role in catalysis or ligand binding and could be promising targets to shorten the human GDE protein while retaining activity.

We first generated 4 GDE mutants with deletion in the C-domain, named ΔC1 to ΔC4, by selecting regions that were less likely to disturb formation of secondary structures, in particular the β-strands mainly composing the C-domain (Supplemental Figure 6D and Supplemental Figure 7A). All expression cassettes containing the mutants were around 5 kb or lower (Figure 1B). Transfection in HEK-293T cells showed that all 4 ΔC mutants were expressed at similar levels (Supplemental Figure 7B). Treatment of *Agl*^–/–^ mice with rAAV vectors encoding the ΔC mutants showed truncated GDE expression and similar VGCN in heart and skeletal muscle of all injected mice (Figure 1C and Supplemental Figure 7, C–E). Importantly, a 50% glycogen reduction was obtained in triceps and quadriceps, suggesting residual activity of the ΔC truncated GDE enzymes, with higher correction achieved in mice treated with rAAV-ΔC1 (Figure 1D, Supplemental Figure 7F, and Supplemental Table 2). Unexpectedly, no correction of glycogen accumulation was observed in the heart of injected *Agl^–/–^* mice (Figure 1D), despite robust tissue transduction and GDE expression. Consistently, a slight improvement of muscle strength assessed by the wire-hang test — a functional test predictive of rAAV gene therapy efficacy (15) — was observed only in mice treated with ΔC1-expressing vector (Figure 1E). These results suggest that, although C-domain–truncated GDE mutants retain some activity, the deletions may affect GDE function in an organ-specific manner, possibly by altering its regulation in the heart.

**Figure 2:**
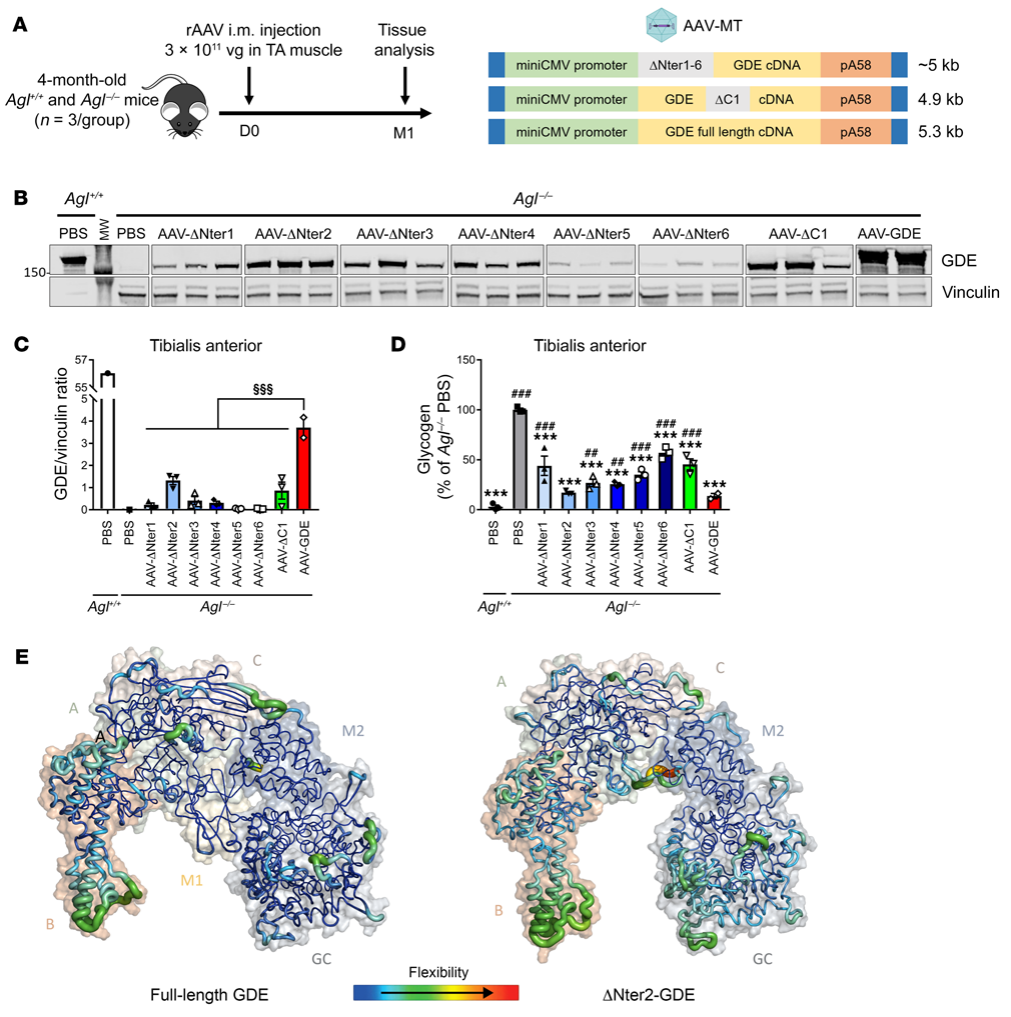
Generation of truncated GDE polypeptides in the N-terminal domain. (**A**) 4-month-old female *Agl*^–/–^ mice were injected in the left tibialis anterior (TA) muscle with an rAAV-MT vector encoding either the human full-length GDE or a truncated GDE, at a dose of 3 × 10^11^ vg/mouse. PBS-injected *Agl*^+/+^ or *Agl*^–/–^ mice were used as controls. (**B**) Western blot analysis of GDE and vinculin expression in the injected TA muscle, 1 month after vector injection. (**C**) Quantification of GDE expression on Western blot showed in panel **B**, expressed as the ratio of the signal of GDE and vinculin bands. (**D**) Glycogen content in the injected TA muscle measured 1 month after vector injection. (**E**) RMSF computed on amino acid residue Cα along the molecular dynamics simulation and projected onto full-length human GDE and ΔNter2-GDE predicted structures using AlphaFold2. Warmer colors and larger ribbons indicate the most fluctuating amino acid residues. Molecular surfaces are shown in transparency with the different domains labelling for clarity purpose. Statistical analyses were performed by 1-way ANOVA. ^§^*P* < 0.05, ^§§^*P* < 0.01, ^§§§^*P* < 0.001 versus *Agl*^–/–^ injected with AAV-GDE in panel **C**; *P < 0.05, **P < 0.01, ***P < 0.001 versus PBS-injected *Agl*^–/–^ mice and ^#^*P* < 0.05, ^##^*P* < 0.01, ^###^*P* < 0.001 versus PBS-injected *Agl*^+/+^ mice in panel **D**; *n* = 3 mice per group. All data are shown as mean ±SEM.

We then generated deletion mutants in the M1 domain, named ΔNter1 to ΔNter6, by selecting regions less likely to disturb the formation of the β-strands composing this domain (Supplemental Figure 8A). After transfection of HEK-293T cells, we observed expression of all the truncated proteins, although ΔNter1, ΔNter2 and ΔNter4 displayed the higher expression, comparable to the ΔC1 mutant (Supplemental Figure 8B). We then evaluated the efficacy of the ΔNter1 to ΔNter6 truncated proteins in *Agl*^–/–^ mice by intramuscular injection in the tibialis anterior muscle, compared with the ΔC1 mutant and the full-length GDE (Figure 2A). Western blot analysis performed in the injected muscle a month after vector injection revealed lower protein expression in all mutants compared with the full-length GDE despite a similar VGCN among the groups (Figure 2, B and C and Supplemental Figure 8C). Of note, the site of truncation had a striking impact on the protein expression level, since ΔNter2-GDE exhibited the highest protein expression, while, in line with our in vitro data, ΔNter5 and ΔNter6 mutants had very low expression (Figure 2, B and C). Importantly, all truncated enzymes displayed some degree of activity. This was reflected by the glycogen reduction in the injected muscle with ΔNter2-GDE showing the highest efficacy, comparable to that of full-length GDE (Figure 2D). We hypothesized that variations in protein expression could be related to differences in protein stability. Indeed, the RNA levels of GDE in the tibialis anterior was similar in mice treated with rAAV encoding either ΔNter2 or full-length GDE, suggesting that the lower ΔNter2 protein levels were likely due to reduced protein stability (Supplemental Figure 8D). Therefore, to further investigate the impact of the ΔNter2 truncation on protein stability, we performed molecular dynamics simulations (250 ns) for both the full-length GDE and the ΔNter2-GDE. Analysis of the conformational variations along the simulation time, monitored by the root mean square deviation (RMSD), revealed a slightly higher flexibility of the truncated protein compared with the parental protein (Supplemental Figure 9A). More detailed inspection of the amino acid fluctuations over the simulation time, described by the root mean square fluctuation (RMSF), showed main differences in the flexibility of the B and GC domains (Figure 2E and Supplemental Figure 9B). Monitoring of the distances between domains along the simulation (Supplemental Figure 9C) suggested that fluctuations did not appear to be directly related to conformational changes inside the domains but rather due to a relative reorientation of the domains. Indeed, distance fluctuations between B and GC domains appeared larger in ΔNter2-GDE than in the full-length protein (Supplemental Figure 9C), likely resulting from A and M2 domains moving apart due to the absence of M1 domain in ΔNter2-GDE. Indeed, the M1 domain is known to maintain the structural integrity of the middle region composed of A, C, and M2 domains (28). Despite of a slightly larger flexibility observed for ΔNter2-GDE compared with the full-length GDE, the structural integrity of the mutant was maintained, and catalytic sites of GC and GT were not affected, indicating that ΔNter2-GDE could be a good candidate for gene therapy.

Oversized rAAV vector production has a large impact on the yields and the quality of the final product, which are critical parameters for the translation to the clinic of rAAV-based gene therapies. To evaluate the impact of the use of the truncated ΔNter2-GDE on the production yields and the quality of the vector, 3 distinct oversized expression cassettes expressing human full-length GDE were compared with the 5 kb expression cassette bearing the ΔNter2-GDE (Supplemental Figure 10A). The use of the ΔNter2-GDE cDNA allowed for approximately 10-fold increase of rAAV production yields (Supplemental Figure 10B) while allowing for the efficient encapsidation of a nontruncated genome (Supplemental Figure 10C). Improved yields and genome quality resulted in a dramatically improved full-to-empty particles ratios, as measured by analytical ultracentrifugation, with up to 37% of full particles measured in rAAV-ΔNter2-GDE (Supplemental Figure 10D).

Taken together, these data indicate that, while ΔNter2-GDE has an in vivo efficacy similar to the full-size enzyme, it allows for production of rAAV vectors with higher yields and quality, thus providing a potential gene therapy candidate for the treatment of GSDIII.

### rAAV encoding ΔNter2-GDE rescues the cardiac and muscle phenotype of Agl^–/–^ mice.

**Figure 3:**
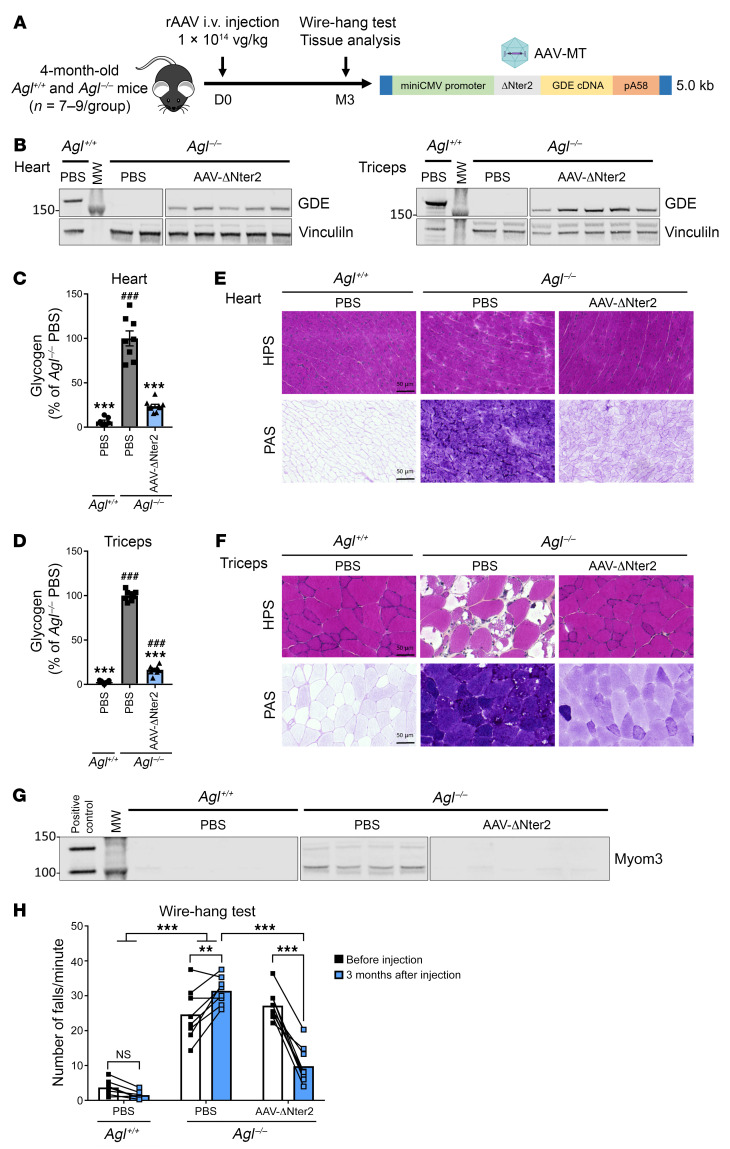
Rescue of muscle and heart impairment in the *Agl*^–/–^ mouse model with rAAV-ΔNter2-GDE vector. (**A**) 4-month-old male *Agl*^–/–^ mice were injected in the tail vein with an rAAV-MT vector encoding ΔNter2-GDE, at the dose of 1 × 10^14^ vg/kg. PBS-injected *Agl*^+/+^ and *Agl*^–/–^ mice were used as controls. (**B**) Western blot analysis of GDE and vinculin expression in heart and triceps, 3 months after vector injection. (**C** and **D**) Glycogen content measured in heart (**C**) and triceps (**D**) 3 months after vector injection. (**E** and **F**) Histological analysis of heart (**E**) and triceps (**F**) using HPS and PAS staining. Representative images are shown (*n* = 7–9). (**G**) Western blot analysis of Myom3 fragments in plasma of mice 3 months after vector injection. Plasma from mdx mouse was used as positive control. (**H**) Wire-hang test expressed as number of falls per minute, performed before and 3 months after vector injection. Statistical analyses were performed by 1-way ANOVA in **C** and **D** and 2-way ANOVA in **H**.*P < 0.05, **P < 0.01, ***P < 0.001 versus PBS-injected *Agl*^–/–^ mice; ^#^*P* < 0.05, ^##^*P* < 0.01, ^###^*P* < 0.001 versus PBS-injected *Agl*^+/+^ mice. *n* = 7–9 mice per group coming from 2 independent experiments. All data are shown as mean ±SEM. Scale bars, 50 μm. HPS, hematoxylin phloxine saffron; Myom3, myomesin 3; PAS, periodic acid schiff.

Next, we evaluated the efficacy of an rAAV-MT vector expressing the ΔNter2-GDE in *Agl*^–/–^ mice via tail vein injection at a dose of 1 × 10^14^ vg/kg (Figure 3A). *Agl*^–/–^ mice were treated at 4 months of age when they showed extensive glycogen accumulation in all muscles and functional impairment as measured by wire-hang (15). Three months after vector injection, VGCN and GDE proteins were detected in all the analyzed muscle tissues (Figure 3B and Supplemental Figure 11, A–C). Glycogen quantification showed almost normalized glycogen levels in the heart and in different skeletal muscles (Figure 3, C and D, Supplemental Figure 11D, and Supplemental Table 3). In line with these data, histological analysis performed on the same tissues showed complete normalization of the muscle architecture on hematoxylin phloxine saffron (HPS) staining as well as an important glycogen reduction by periodic acid-schiff (PAS) staining (Figure 3, E and F and Supplemental Figure 12, A–C). We also evaluated by Western blot the levels of myomesin3 (Myom3) fragments, a known biomarker of muscle dystrophies (39). Although GSDIII is not a muscular dystrophy, we found that Myom3 was elevated in the plasma of *Agl*^–/–^ mice at baseline. It normalized 3 months after treatment in mice injected with rAAV encoding for ΔNter2-GDE (Figure 3G and Supplemental Figure 12, D and E). Importantly, treatment with rAAV-ΔNter2-GDE rescued muscle strength assessed by the wire hang test. Before injection, *Agl*^–/–^ animals exhibited a high frequency of falls (26.0 falls/min ± 1.4), compared with *Agl*^+/+^ mice (3.7 falls/min ± 0.8) (Figure 3H). Three months after injection, rAAV-ΔNter2-GDE–injected mice showed less frequency of falls compared with the PBS-injected group (9.8 falls/min ± 1.8 versus 31.4 falls/min ± 1.4), almost reaching WT levels (Figure 3H). To confirm these results, muscle strength was also evaluated 5 months after injection of rAAV-ΔNter2 in another cohort of *Agl*^–/–^ mice, using an extended set of functional evaluations (Supplemental Figure 13A). Improvement of muscle strength was observed in rAAV-ΔNter2-treated animals by both the wire hang and the grip strength tests (Supplemental Figure 13, B and C). However, the rotarod test performances were not impaired in *Agl*^–/–^ mice and remained unchanged in treated animals (Supplemental Figure 13D). Finally, we confirmed the strong liver detargeting by analyzing the liver of *Agl*^–/–^ mice treated with rAAV-ΔNter2-GDE. Considering the dose used, low VGCN were observed in the liver of *Agl*^–/–^ mice 3 months after injection, with no detectable GDE expression (Supplemental Figure 14, A and B). Therefore, no correction of the liver weight, accumulation of glycogen, glycemia, or of the aspartate and alanine aminotransferases levels in plasma was observed (Supplemental Figure 14, C-F). Liver histology was similar between untreated and treated *Agl*^–/–^ mice, with similar levels of fibrosis (Supplemental Figure 14, G and H). These data clearly demonstrate that treatment with rAAV-ΔNter2-GDE reverses the muscle impairment in adult, symptomatic GSDIII mice at both biochemical and functional levels, in the absence of liver targeting.

### rAAV encoding ΔNter2-GDE corrects glycogen accumulation in an Agl^–/–^ rat model of GSDIII.

**Figure 4:**
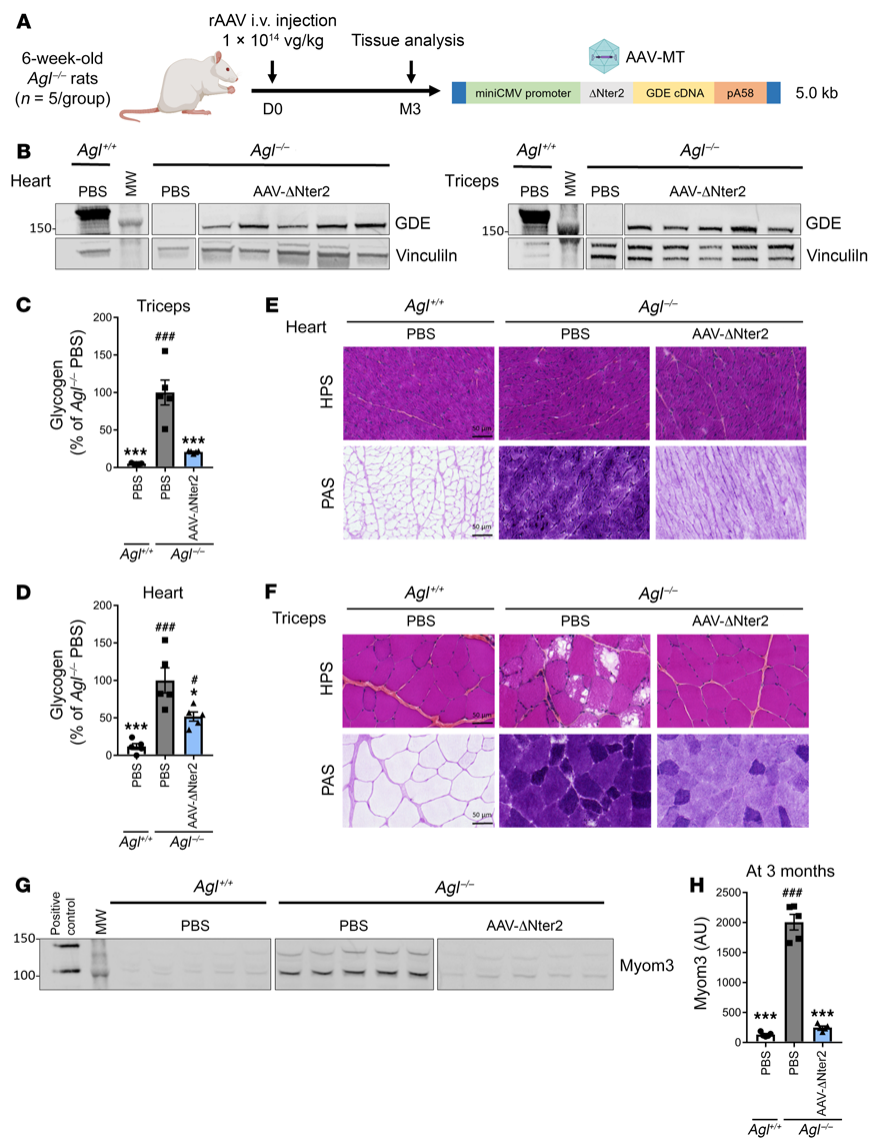
Correction of muscle and heart impairment in the *Agl*^–/–^ rat model with rAAV-ΔNter2-GDE vector. (**A**) 6-week-old male *Agl*^–/–^ rats were injected in the tail vein with an rAAV-MT vector encoding the ΔNter2-GDE, at the dose of 1 × 10^14^ vg/kg. PBS-injected *Agl*^–/–^ and *Agl*^+/+^ rats were used as controls. (**B**) Western blot analysis of GDE and vinculin expression in heart and triceps, 3 months after vector injection. (**C** and **D**) Glycogen content measured in triceps (**C**) and heart (**D**) 3 months after vector injection. (**E** and **F**) Histological analysis of heart (**E**) and triceps (**F**) with HPS and PAS staining. Representative images are shown (*n* = 5). (**G**) Western blot analysis of Myom3 fragments in the plasma of rats 3 months after vector injection. Plasma from mdx mouse was used as positive control. (**H**) Quantification of Myom3 expression on the Western blot showed in Panel **G** (arbitrary units). Statistical analyses were performed by 1-way ANOVA. *P < 0.05, **P < 0.01, ***P < 0.001 versus PBS-injected *Agl*^–/–^ rats; ^#^*P* < 0.05, ^##^*P* < 0.01, ^###^*P* < 0.001 versus PBS-injected *Agl*^+/+^ rats; *n* = 5 rats per group. All data are shown as mean ±SEM. Scale bars, 50 μm. HPS, hematoxylin phloxine saffron; Myom3, myomesin 3; PAS, periodic acid schiff.

To confirm the efficacy of the optimized rAAV-ΔNter2-GDE in a larger animal model, we administered the vector to 6-week-old *Agl*^–/–^ rats. *Agl*^–/–^ rats, treated with 1 × 10^14^ vg/kg of rAAV-ΔNter2-GDE via tail vein injection, were analyzed 3 months after injection (Figure 4A). Vector genomes and ΔNter2-GDE proteins were detected in all evaluated muscles (Figure 4B and Supplemental Figure 15, A–C). Treatment with rAAV-ΔNter2-GDE reduced glycogen content in different skeletal muscles to levels close to those measured in *Agl*^+/+^ rats. (Figure 4C, Supplemental Figure 15, D–F, and Supplemental Table 4). A significant, 50%, glycogen reduction was observed in the heart (Figure 4D), which was even more evident in the PAS staining (Figure 4E). Histological analysis also showed normalization of muscle architecture, as well as an important glycogen reduction in all skeletal muscles tested (Figure 4F, Supplemental Figure 15, G–I, and Supplemental Figure 16, A and B). Analysis of the Myom3 levels in 6-week-old *Agl*^–/–^ rats showed moderate elevation compared with age-matched controls (Supplemental Figure 16, C and D). Importantly, *Agl*^–/–^ rats treated with rAAV-ΔNter2-GDE showed reduced Myom3 levels, similar to *Agl*^+/+^ rats, 3 months after vector injection (Figure 4, G and H). Acute cardiac toxicity has already been reported following high-dose rAAV-based gene therapy targeting the heart in animal models and in patients (40, 41). No death, weight loss, or clinical signs of acute toxicity were reported in treated rats. Quantification of plasma cardiac troponin I and T (cTnI and cTnT) and of the N-terminal fragment of the probrain natriuretic peptide (NT-pro-BNP) levels, clinically relevant markers of cardiac damage and heart failure, respectively, did not reveal any difference between PBS-injected and rAAV-ΔNter2-GDe–injected animals 1 month and 3 months after injection (Supplemental Figure 16E).

In conclusion, the data obtained by the treatment of *Agl*^–/–^ rats with an rAAV vector encoding for ΔNter2-GDE confirm those obtained in the mouse model of the disease and further support the clinical translation of rAAV-ΔNter2-GDE to treat the muscle disease in patients with GSDIII.

### ΔNter2-GDE reduces glycogen accumulation in a human skeletal muscle cell model of GSDIII.

**Figure 5:**
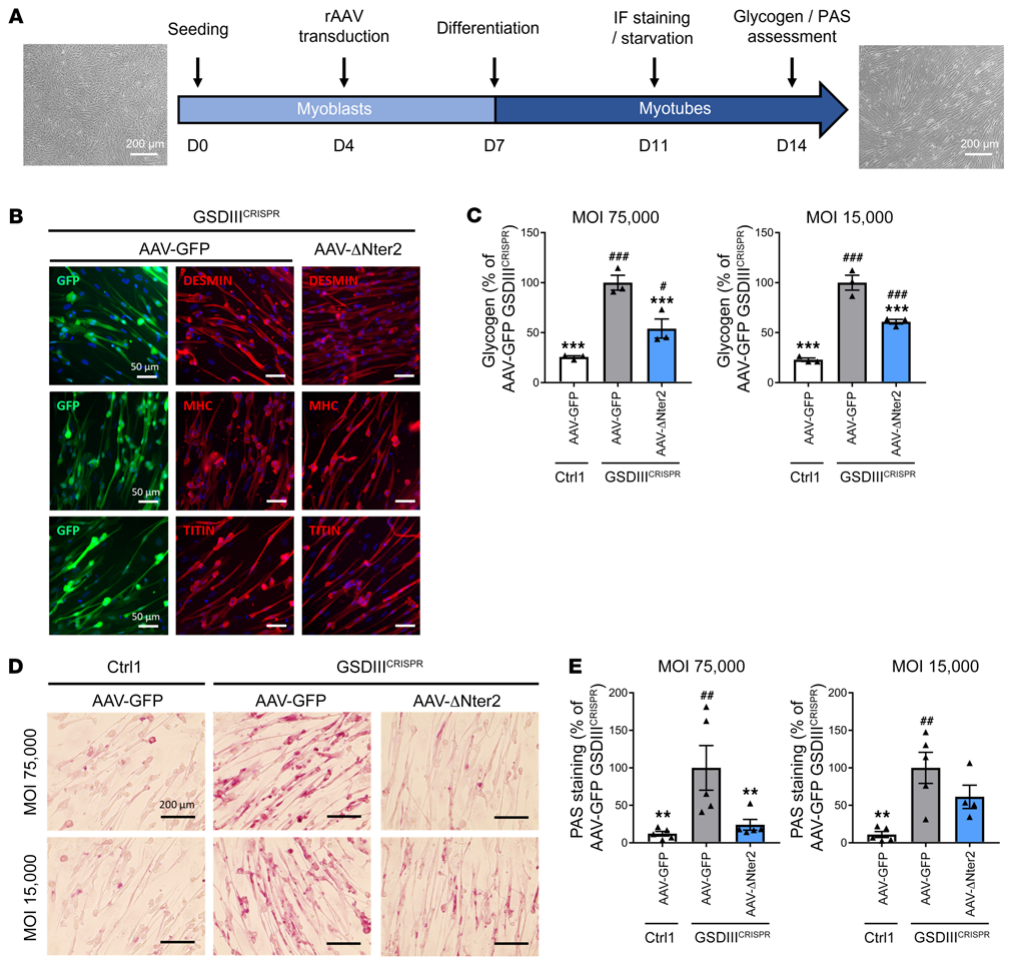
Glycogen reduction in hiPSC-derived muscle cells treated with rAAV-ΔNter2-GDE. (**A**) Transduction protocol of GSDIII^CRISPR^ hiPSC-derived skMb and isogenic controls with an rAAV-LK03 vector expressing either GFP or ΔNter2-GDE at a MOI of either 15,000 or 75,000. Scale bars, 200 μm. (**B**) Expression of specific myogenic markers (desmin, myosin heavy chain [MHC] and titin) and GFP by immunofluorescence analysis, after rAAV transduction of GSDIII^CRISPR^ hiPSC-derived skMt at a MOI of 75,000. Scale bars, 50 μm. Representative images are shown (**C**) Glycogen content normalized to the number of viable cells measured in GSDIII^CRISPR^ hiPSC-derived skMt and isogenic controls (Ctrl1) transduced as described above. (**D**) PAS staining on GSDIII^CRISPR^ hiPSC-derived skMt and isogenic controls (Ctrl1) transduced as described above. Representative images are shown. Scale bars, 200 μm. (**E**) Quantification of PAS staining. Statistical analyses were performed by 1-way ANOVA. *P < 0.05, **P < 0.01, ***P < 0.001 versus GSDIII^CRISPR^ cells transduced with rAAV-GFP; ^#^*P* < 0.05, ^##^*P* < 0.01, ^###^*P* < 0.001 versus CTRL1 cells transduced with rAAV-GFP, 3 replicates for experiments in panel **B** and **C** and 5 replicates for experiments in panel **D** and **E**. All data are shown as mean ±SEM. PAS, periodic acid Schiff; skMb, skeletal myoblasts; skMt, skeletal myotubes.

To evaluate the activity of the truncated ΔNter2-GDE in a human pathological context, we took advantage of a recently reported in vitro human skeletal muscle model of GSDIII derived from human induced pluripotent stem cells (hiPSCs) edited by CRISPR/Cas9 technology (GSDIII^CRISPR^) (42). Skeletal muscle cells derived from the isogenic control hiPSC line (CTRL1) were used as an unaffected control (42). We produced an rAAV vector expressing ΔNter2-GDE or GFP suitable for in vitro use and we treated GSDIII^CRISPR^ and CTRL1 hiPSC-derived skeletal myoblasts (skMb) following a recently reported protocol (42) (Figure 5A). After transduction with rAAV, skMb were differentiated into skeletal myotubes (skMt). Transduction with rAAV expressing GFP or ΔNter2-GDE did not alter the differentiation of skMb, that showed similar expression of skeletal myogenic markers by immunostaining analysis (Figure 5B and Supplemental Figure 17A). Cell viability was reduced in GSDIII^CRISPR^ skMt compared with CTRL1 skMt and was similar between GFP- and ΔNter2-GDetransduced cells (Supplemental Figure 17B). GSDIII^CRISPR^ skMt transduced with rAAV expressing ΔNter2-GDE exhibited a significant reduction of glycogen content when compared with GSDIII^CRISPR^ skMt transduced with the control vector (Figure 5C). PAS staining performed on transduced-skMt confirmed these results (Figure 5, D and E). These data demonstrate that the truncated ΔNter2-GDE reduced glycogen accumulation not only in mouse and rat muscles, but also in a human skeletal muscle model of GSDIII.

## Discussion

In GSDIII, inactivating mutations on the *AGL* gene result in the absence of a functional GDE and accumulation of limit dextrin in liver, heart, and skeletal muscles. The hepatic and metabolic phenotypes (hepatomegaly and hypoglycemia) appear early, in patients of around 1 year of age (2). Moreover, recent publications report that children exhibit liver fibrosis as early as 1 year of age (6). Although hypoglycemia and liver enzymes usually improve with age, the liver disease progresses and some adult patients present with cirrhosis and tumors and may require liver transplantation (2, 3). Nonetheless, the major disease burden at adulthood is the heart and muscle phenotype, with up to 80% of patients presenting with muscle weakness and one-third with loss of ambulation (2, 3). Whereas dietary treatment helps to control the hypoglycemic episodes, it has little effects on the peripheral myopathy (1, 7). In the absence of a curative treatment for GSDIII, the muscle and heart manifestations remain as the most critically unmet medical needs. Gene therapy with rAAV vectors appears as an attractive option to correct the muscle and heart phenotype of GSDIII (14), but the size of the GDE cDNA (4.6 kb) along with the required regulatory sequences prevents its efficient encapsidation in a single rAAV. Two different strategies have been published, each with their own drawbacks: a dual vector approach (15) and a pullulanase-based approach (20, 21). While the dual vector approach was efficient for muscle and heart correction (15), it required 2 vectors and might expose patients to toxicities. The second strategy relied on a single vector encoding the bacterial enzyme pullulanase and induces immune response toward the transgene (21). In the present work, we used rational engineering to generate a truncated version of the human GDE efficiently packaged in a single rAAV vector, intended for the correction of the cardiac and muscular manifestations of the disease in adult patients. Two arguments supported the rationale for liver detargeting, achieved by the use of a recently developed engineered AAV capsid (24): first, the correction of the liver disease in GSDIII at adulthood is challenging in preclinical models, likely due to hepatocyte proliferation and liver fibrosis, as previously reported (15). In particular, incomplete and heterogeneous correction was achieved after injection of a liver-optimized high-dose dual rAAV vector (15). Human patients also present with fibrosis starting from young ages (6) and might require an early treatment in childhood before marked fibrosis has developed. In this setting, rAAV would not be the first-line option, because of potential rAAV dilution after liver growth. Second, and most importantly, high-dose gene therapy clinical trials for neuromuscular disease have revealed the presence of severe, sometimes lethal, liver-associated toxicities, especially in the presence of an underlying liver disease (22, 23). Because of the presence of liver fibrosis in GSDIII early in life, as well as cirrhosis and portal hypertension in a third of adult patients (2, 3, 6), we decided to detarget the liver to address the main disease manifestations during adulthood while ensuring safety.

A translationally viable strategy to achieve efficient rAAV packaging and broader muscle cell transduction with large transgenes is the generation of shorter proteins (43, 44). A similar approach has also been successfully used for Wilson disease (45). GDE is a complex enzyme that involves 2 distinct enzymatic activities in a single polypeptide chain (28). As such, even slight modifications of the sequence may induce a dramatic reduction of the activity. To reduce the size of GDE, we took advantage of the large number of reported missense variants in patients with GSDIII. We identified 2 putative regions for which we derived different truncated candidates. Intriguingly, deletion mutants within the C domain displayed activity in skeletal muscles, but not in the heart. Further characterization of the underlying mechanisms is ongoing although the working hypothesis is that the C domain participates in the regulation of GDE activity in a tissue-specific manner. Different from the C-domain mutants, the N-terminal–truncated ΔNter2-GDE was active and decreased glycogen accumulation in both heart and skeletal muscle. This truncation was predicted by molecular modelling to have limited impact on the structural integrity of the protein and its catalytic sites. However, molecular dynamics simulations indicated a higher flexibility of ΔNter2-GDE compared with the full-length GDE, which is coherent with the lower protein stability observed experimentally. The use of molecular dynamics simulations is therefore a valuable tool for the design of truncated proteins to enable rAAV gene therapy in diseases that involve large transgenes.

The use of ΔNter2-GDE allowed us to generate a potent, high-quality product for GSDIII. rAAV-ΔNter2-GDE corrected muscle and heart phenotypes in both mouse and rat models of GSDIII with extensive glycogen reduction, reflecting broader cell transduction. Treatment of *Agl^–/–^* mice with rAAV-ΔNter2-GDE vector promoted the recovery of muscle strength 3 months after injection. This suggests that in GSDIII, the myopathy — which displays low fibrosis and muscle fiber renewal, in contrast to what has been observed in muscular dystrophies (5, 46) — can be reversed, at least in rodent models.

A recently developed human skeletal muscle model of GSDIII derived from hiPSCs edited by CRISPR/Cas9 technology (42) offers a unique opportunity to evaluate the efficacy and safety of the expression of the truncated ΔNter2-GDE in a human pathological context. These cells, in contrast to explanted cells, have self-renewal capacities and the possibility of differentiation in a variety of cell types (47). In the context of GSDIII, the limited access to liver and muscle biopsies makes in vitro human models of the affected tissues essential to evaluate safety and efficacy of therapeutic approaches. In our study, we demonstrated that ΔNter2-GDE clears the accumulated glycogen, suggesting that the truncation on the N-terminal region does not affect the enzyme function and regulation in a human cellular context. Furthermore, cell viability and myogenic markers were similar between rAAV-GFP- and rAAV-ΔNter2-transduced cells, suggesting the absence of specific alteration related to the expression of the truncated mutant in human muscle cells. These data are extremely valuable in the prospect of translating this gene therapy approach into humans.

Beyond the correction of the muscle disease, further optimizations could improve single rAAV vector gene transfer for GSDIII. Targeting of the liver may prove complex due to the underlying liver disease (2, 3, 6) and the complexity of developing AAV capsids able to target the liver without overloading at the doses used for muscle gene transfer. However, the use of a muscle and liver tropic capsid combined with a short promoter able to achieve both liver and muscle transgene expression could potentially allow complete correction of GSDIII phenotype. Indeed, tandem promoters with both efficient liver and muscle expression have been described, but are too large to be efficiently encapsidated along with the ΔNter2-truncated GDE in a single rAAV vector (18, 21). Combination of smaller promoters and novel rAAV capsids with a liver-muscle targeting tailored to GSDIII may be used in the next future to improve the safety and efficacy of the approach. Another potential approach to provide full rescue of the disease phenotype would be the combination of our approach with mRNA expressing the transgene directly in hepatocytes, as recently reported for other metabolic diseases (48).

In conclusion, our work provides proof-of-concept of the use of a single rAAV vector expressing a truncated form of GDE for the rescue of muscle and heart impairment in 2 GSDIII rodent models as well as in a recently described human muscle cell model of GSDIII, which supports the clinical translation of this approach to provide a one-shot, definitive treatment for this burdensome disease.

## Methods

### In vivo studies.

The *Agl* knock-out mice (*Agl*^–/–^, *Agl*^tm1b(EUCOMM)Wtsi^) were previously described (15) and are of mixed background (C57BL/6J and Balb/c). The *Agl* knock-out rats (*Agl*^–/–^) were recently generated using the CRISPR tool and are of Sprague Dawley background. rAAV vectors encoding human GDE were either i.v. administered via the tail vein to 4-month-old male *Agl*^–/–^ mice and WT littermates (*Agl*^+/+^) or intramuscularly in the tibial anterior muscle of 4-month-old female *Agl*^–/–^ mice and *Agl*^+/+^ WT littermates. Intramuscular injection was performed in the tibialis anterior muscle under anesthesia by ketamine and xylazine. rAAV vectors encoding GDE were also administered in the tail vein of 6-week-old male *Agl*^–/–^ rats and *Agl*^+/+^ WT littermates. Finally, rAAV vectors encoding mSEAP or Luciferase were i.v. administered via the tail vein to 6-week-old WT male C57BL/6J mice. All animals were randomized to receive rAAV or PBS as controls. Mice were euthanized by cervical dislocation and rats were sacrificed by anesthetic overdose.

### Production of rAAV vectors.

All rAAV vectors used in this study were produced using an adenovirus-free transient transfection method and purified using a chromatographic method as described earlier (49). Titers of the rAAV vector stocks were determined using a real-time quantitative PCR using primers for the codon-optimized GDE cDNA (forward: 5′-CTG AAG CTG TGG GAG TTC TT-3′ and reverse: 5′-CTC TTG GTC ACT CTT CTG TTC TC-3′) or for ITRs (forward: 5′-GGA ACC CCT AGT GAT GGA GTT-3′ and reverse: 5′-CGG CCT CAG TGA GCG A-3′), for vectors encoding mSEAP or Luciferase. The mSEAP expression cassette contains the specified promoter, the SV40 intron, the mSEAP cDNA, and an SV40 poly A signal. The Luciferase expression cassette contains the CMV promoter, the luciferase cDNA, and an SV40 poly A signal. The GDE expression cassette contains the mini CMV promoter (corresponding to the nucleotides 175050_175400 of the CMV genome, NC_006273), the human full-length or truncated codon-optimized GDE cDNA (15, 50), and either a bGh or a pA58 poly A signal (43). All cassettes were flanked by the ITRs of AAV serotype 2 for vector packaging. The capsid used (AAV-MT) is a hybrid between AAV9 and AAVrh74, harboring a P1 peptide, as previously described (24).

### Analytical ultracentrifugation.

Analytical ultracentrifugation measures the sedimentation coefficient of macromolecules by following over time the optical density of a sample subjected to ultracentrifugation. The difference in the sedimentation coefficient, measured by Raleigh interference or 260-nm absorbance, depends on the content of viral genome in the capsid. Analysis was performed using a Proteome Lab XL-I (Beckman Coulter). An aliquot of 400 μL rAAV vector and 400 μL formulation buffer were loaded into a 2-sector velocity cell. Sedimentation velocity centrifugation was performed at 20,000 *g* and 20°C. Absorbance (260 nm) and Raleigh interference optics were used to simultaneously record the radial concentration as a function of time until the lightest sedimenting component cleared the optical window (approximately 1.5 hours). Absorbance data required the use of extinction coefficients to calculate the molar concentration and the percent value of the empty and genome-containing capsids. Molar concentrations of both genome-containing and empty capsids were calculated using Beer’s law, and percentages of full genome-containing and empty capsids were calculated.

### Viral genome analysis on agarose gel.

DNA was extracted using the High Pure Viral Nucleic Acid Kit (Roche). Purified viral DNA was then loaded on a 1% agarose gel (Eurobio Scientific) stained with SybrSafe Gel Stain (Invitrogen) to visualize the viral DNA.

### Western blot analysis.

Mouse and rat tissues were homogenized with FastPrep lysis tubes (MP Biomedicals) in PBS with cOmplete protease inhibitor cocktail (Roche). Protein concentration was determined using the Pierce BCA Protein Assay (Thermo Fisher Scientific) according to the manufacturer’s instructions. A fraction of 50 μg of total proteins were loaded in each well for both PBS- and rAAV-injected *Agl*^–/–^ mice and rats and 10 μg of total proteins per well for PBS-injected *Agl*^+/+^ mice and rats. SDS-PAGE electrophoresis was performed in a 4%–12% Bis-Tris gradient polyacrylamide gel (NuPAGE, Invitrogen). After transfer, the membrane was blocked with Intercept Blocking buffer (LI-COR Biosciences) and incubated with either an anti-GDE rabbit polyclonal antibody (16582-1-AP, Proteintech for muscles or AS09454, Agrisera for liver; 1:1,000) and an anti-vinculin mouse monoclonal antibody (V9131, Sigma-Aldrich) for tissue lysates and with an anti-Myom3 rabbit polyclonal antibody (17692-1-AP, Proteintech, 1:1,000) for plasma. The membrane was washed and incubated with the appropriate secondary antibody (LI-COR Biosciences, 1:10,000) and visualized by Odyssey imaging system (LI-COR Biosciences). For in vitro experiments, normalization was performed using an anti-actin mouse monoclonal antibody (66009-1-Ig, Proteintech, 1:1,000). When the number of samples required the use of two gels, both were processed in parallel, including running and transfer within the same tank, incubation with the same antibody solution, and visualization at the same time by the Odyssey imaging system.

### HEK-293T cells transfection.

HEK 293T cells were transfected in 6-well plates using the Opti-MEM medium and Lipofectamine 3000 transfection reagent (Thermo Fisher Scientific) according to the manufacturer’s instructions. Cells were harvested 48 hours after transfection and lysed in PBS with 1% Triton X-100. Supernatants were collected after centrifugation at 11,000*g* and 50 μg of total proteins were used for Western blots.

### Measurement of glycogen content in tissues.

Glycogen content was measured indirectly in tissue homogenates as the glucose released after total digestion with *Aspergillus niger* amyloglucosidase (Sigma-Aldrich). Samples were incubated for 10 minutes at 95°C and then cooled at 4°C. After the addition of amyloglucosidase (final concentration 4 U/mL) and potassium acetate pH 5.5 (final concentration 25 mM) at 37°C for 90 minutes, the reaction was stopped by incubating samples for 10 minutes at 95°C. A control reaction without amyloglucosidase was prepared for each sample and was incubated in the same conditions. The glucose released was determined using a glucose assay kit (Sigma-Aldrich) and the resulting absorbance was acquired on an EnSpire Alpha plate reader (PerkinElmer) at a wavelength of 540 nm. Glucose released after amyloglucosidase was then normalized by the total protein concentration.

### Measurement of glycemia and plasma aspartate aminotransferase and alanine aminotransferase levels.

Blood samples were taken from mice anesthetized with isoflurane. Glycemia was measured using a glucometer (Accu-Chek). Aspartate and alanine aminotransferase levels were measured on plasma using micro-chip DRI-CHEM SLIDE (Fujifilm, AST-P III, ALT-P III) and DRI-CHEM NX500 spectrophotometer (Fujifilm) following the manufacturer’s instructions.

### Measurement of cardiac troponin and NT-pro-BNP levels in rat plasma.

Quantification was performed following the manufacturer’s instructions at the minimal possible plasma dilution (i.e., 1:2), using the Meso Scale Discovery (MSD) ELISA Rat Cardiac Injury Panel 2 Kit (ref K15155) and Rat NT-pro-BNP Assay Kit (ref K153JKD).

### Histology.

Heart, triceps brachii, quadriceps femoris, soleus, EDL, and liver were snap frozen in isopentane previously chilled in liquid nitrogen. Serial 8-μm cross sections were cut in a Leica CM3050 S cryostat (Leica Biosystems). To minimize sampling error, 2 sections of each specimen were obtained and stained with HPS, PAS, and/or Sirius Red (SR) according to standard procedures. Images were digitalized using Axioscan Z1 slide scanner (Zeiss) under a Zeiss Plan-Apochromat 10X/0.45 M27 dry objective (Zeiss). Tile scan images were reconstructed with ZEN software (Zeiss). Quantification of images were processed using QuPath 0.4.3 Software (51). For the PAS staining, a first pixel classifier was trained on different types of muscle slices for detecting tissue and eliminating artefacts such as folding, bubbles, and tearings. For mice, this PAS contour pixel classifier is a Random Tree with a resolution of 14.08 μm/pixel, includes 3 channels, 5 scales (0.5, 1, 2, 4, and 8), 8 features (Gaussian, Laplacian of Gaussian, weighted deviation, gradient magnitude, structure tensor max eigenvalues, structure tensor middle eigenvalues, structure tensor min eigenvalues, and structure tensor coherence), and no local normalization. The quantification of PAS staining was performed using a pixel classifier trained on healthy tissue and impaired tissue. The output parameter is then the ratio of the surface area of impaired tissue over the surface area of the total tissue slice (obtained by the contour pixel classifier). The PAS quantification classifier is an Artificial Neural Network with a resolution of 1.76 μm/pixel (for rats) or 3.51 μm/pixel (for mice), includes 3 channels, 4 scales (0.5, 1, 2, and 4), 6 features (Gaussian, Laplacian of Gaussian, weighted deviation, gradient magnitude, structure tensor max eigenvalues, and structure tensor middle eigenvalues), and no local normalization. For the HPS staining, a pixel classifier was trained on different types of muscle slices for detecting tissue and eliminating artefacts such as folding, bubbles, and tearings. This HPS contour pixel classifier is a Random Tree with a resolution of 14.08 μm/pixel, includes 3 channels, 3 scales (2, 4, 8), 4 features (Gaussian, Laplacian of Gaussian, weighted deviation, and gradient magnitude), and no local normalization. The quantification was performed using a pixel classifier trained on healthy tissue and impaired tissue. The output parameter is then the ratio of the surface area of impaired tissue over the surface area of the total tissue slice (obtained by the contour pixel classifier). The HPS quantification classifier is an Artificial Neural Network with a resolution of 7.03 μm/pixel, includes 3 channels, 5 scales (0.5, 1, 2, 4, and 8), 5 features (Gaussian, Laplacian of Gaussian, weighted deviation, gradient magnitude, and structure tensor max eigenvalues), and no local normalization. For the quantification of fibrosis, the pixel classification feature from QuPath (51) 0.4.3 was used by creating 2 classifiers, using each time 2 images for ground truth. The first pixel classifier identifies pixels belonging to the tissue slice, excluding veins, fold, dust, bubbles, or any artifact encountered, and draw an annotation of the analyzable tissue. The second classifier was trained to identify fibrosis and healthy tissue, based on manual annotation of picrosirius red staining. The fibrosis classifier was then applied in the annotation created by the first classifier. The total surface of SR staining was divided by the total surface area of the muscle slice resulting then in a fibrosis ratio (% of fibrotic tissue) for each tissue slice.

### Vector genome copy number determination.

Vector genome copy number was determined using a quantitative PCR assay as previously described (27). The PCR primers used in the reaction were located in the glucosyltransferase domain of the full-length and truncated codon-optimized GDE (forward: 5′-CTG AAG CTG TGG GAG TTC TT-3′ and reverse: 5′-CTC TTG GTC ACT CTT CTG TTC TC-3′) or in the ITRs (forward: 5′-GGA ACC CCT AGT GAT GGA GTT-3′ and reverse: 5′-CGG CCT CAG TGA GCG A-3′) for mSEAP expressing cassettes. As an internal control, primers located within the mouse (forward: 5′-AAA ACG AGC AGT GAC GTG AGC-3′ and reverse: 5′-TTC AGT CAT GCT GCT AGC GC-3′) or rat (forward: 5′-AAA ACG AGC GGT GAC ATG AGC-3′ and reverse: 5′-TTC AGT CAT GCT AGC GCT CC-3′D) *Titin* gene were used.

### RNA expression analysis.

Total RNAs were extracted from cell lysates using Trizol (Thermo Fisher Scientific) and the RNeasy Mini Kit (Qiagen). DNA contaminants were removed using the Free DNA kit (Thermo Fisher Scientific). Total RNAs were reverse transcribed using random hexamers and the RevertAid H minus first strand cDNA synthesis kit (Thermo Fisher Scientific). Quantitative PCR was performed with oligonucleotides specific for the codon-optimized GDE transgene (forward: 5′-CTG AAG CTG TGG GAG TTC TT-3′ and reverse: 5′-CTC TTG GTC ACT CTT CTG TTC TC-3′) and normalized by the levels of expression of the P0 ribosomal protein *Rplp0* mRNA (forward: 5′-CTC CAA GCA GAT GCA GCA GA-3′; reverse: 5′-ATA GCC TTG CGC ATC ATG GT-3′).

### mSEAP quantification.

mSEAP was quantified in tissue lysates using the Phospha-Light Kit (Applied Biosystems) following the manufacturer’s instructions and was normalized by the total protein concentration measured using the Pierce BCA Protein Assay (Thermo Fisher Scientific).

### Luciferase quantification.

Snap-frozen tissues were homogenized in PBS with FastPrep lysis tubes (MP Biomedicals), followed by centrifugation at 10,000 *g* for 10 minutes. Supernatants were collected and diluted in lysis buffer (1 mM DTT, 25 mM Tris/base, 1 mM EDTA, 8 mM MgCl_2_, 15% glycerol, and 0.4% Triton [Sigma Aldrich]) in a white opaque 96-well plate (PerkinElmer). Luciferase activity was measured using EnSpire (PerkinElmer) through sequential injections of assay buffer (1 mM DTT, 25 mM Tris/base, 1 mM EDTA, 8 mM MgCl_2_, 15% glycerol, and 2 mM ATP [Sigma Aldrich]) and luciferine (Interchim). Luciferase relative luminescence unit was normalized by the total protein concentration measured using the Pierce BCA Protein Assay (Thermo Fisher Scientific).

### Muscle function.

A forelimb wire-hang test was performed as already reported (52, 53) at baseline and each month until euthanasia. A 4-mm-thick wire was used to record the number of falls over a period of 3 minutes. The average number of falls per minute was reported for each animal. Grip strength and Rotarod tests were performed as previously reported (15).

### Transduction of hiPSC-derived skeletal muscle cells.

The GSDIII^CRISPR^ hiPSCs have been previously generated, using CRISPR knock down of the *AGL* gene (42). Control hiPSCs were the isogenic cell line (CTRL1). GSDIII^CRISPR^ and CTRL1 hiPSCs were differentiated into skMb, as previously described (42). After expansion in 96-well plate, hiPSC-derived skMb were transduced with LK03-rAAV vectors encoding either GFP or ΔNter2-GDE under the control of the miniCMV promoter, at a multiplicity of infection (MOI) of either 75,000 or 15,000 for 72 hours. Then, hiPSC-derived skMb were differentiated into skMt, as previously described (42).

### Measurement of Glycogen Content in skMt.

After 4 days of differentiation into skMt, hiPSC-derived skMt were starved for 3 days in a no-glucose DMEM medium with 10% FBS (Thermo Fisher Scientific) in order to induce glycogen degradation in CTRL1 skMt as previously described (42). Cells were lysed using HCl 0.3M and Tris 450 mM pH 8.0. Glycogen was then quantified using the Glycogen-Glo assay kit (Promega) and normalized using the CellTiter-Glo Luminescent Cell Viability Assay (Promega).

### Immunostaining assay.

SkMt derived from hiPSCs were fixed with 4% paraformaldehyde (Euromedex) for 10 minutes at room temperature. After 2 washes in PBS, cells were permeabilized with 0.5% Triton X-100 for 5 minutes and blocked in PBS solution supplemented with 1% BSA (Sigma-Aldrich) for 1 hour at room temperature. SkMt were stained for specific skeletal myogenic markers overnight at 4°C using primary antibodies (Desmin, ref AF3844 R&D 1:200; MHC/MF20, ref 3ea DSHB 1:50; Titin ref T5650 US Biological 1:50). After 3 washes in PBS, staining was revealed by appropriate Alexa Fluor secondary antibodies 1:1,000 (Donkey anti-goat AF488, ref A11055 Invitrogen 1:1,000; Donkey anti-mouse AF488, ref A21202 Invitrogen 1:1,000) in the dark for 1 hour at room temperature, and nuclei were visualized with Hoechst solution 1:3,000 (Invitrogen). Cell imaging was carried out with a Zen Black software-associated LSM-800 confocal microscope (Zeiss) with a 20× objective.

### PAS staining on skMt.

PAS staining on hiPSC-derived skMt was performed with the PAS Staining Kit (Sigma-Aldrich) following the manufacturer’s instructions. Briefly, cells were fixed with 4% paraformaldehyde for 10 minutes at room temperature. After 2 washes in PBS, cells were treated with PAS for 5 minutes at room temperature. After 3 washes in distilled water, cells were treated with Schiff’s reagent for 15 minutes at room temperature. Finally, after 4 washes in tap water, staining was visualized using an EVOS XL Core microscope (Invitrogen). Images were processed and analyzed using FIJI custom-made scripts (54). First, colors were split and only the green channel was kept as it was the most contrasted channel. Images were manually thresholded into binary images where PAS signal was black and background white. The threshold was set for maximizing the difference between genotypes, and, once calculated, the same threshold was applied to all images to quantify. The quantification of PAS staining was obtained using this formula: Area of PAS staining / Total area of image × 100, giving a percentage of PAS staining within the image.

### Molecular modelling.

A three-dimensional model of the full-length human GDE was retrieved from the AlphaFold2-database (30). The 3D model of ΔNter2-GDE was predicted using AlphaFold2 v.2.1 (29) and the default parameters. Molecular dynamics simulations were performed using Gromacs 2021.3 (55) with CHARMM36 forcefield (56). Solvation was done using explicit TIP3 water model. A cut-off of 1.2 nm and a switch function from 1.0 to 1.2 nm were used for short-range electrostatic and van der Waals interactions, respectively. Long-range electrostatic interactions were treated with particle mesh Ewald (parameters by default) and periodic boundary conditions. The following protocol was used: (a) minimization of the system with 50,000 steps of steepest descent algorithm, (b) 100 ps of NVT equilibration, (c) 100 ps of NPT equilibration, (d) 250 ns production phase without constraints and a timestep of 2 fs. molecular dynamics simulations were run at 303 K in triplicates. To analyze molecular dynamics trajectories, we used the molecular dynamics analysis packages (57). The RMSD was computed using the Cα atoms along the simulation, RMSF per amino acid residue were calculated using the residue Cα atoms along the simulation and the inter-domain distances were measured between the center of mass of the different domains. Structural images were prepared using Pymol2.5 (Schrodinger Inc.).

### Statistics.

All the data shown in this paper are reported as mean ± SEM. The GraphPad Prism software was used for statistical analysis. *P* values < 0.05 were considered significant. For all the data sets, data were analyzed by parametric tests, α = 0.05 (1-way and 2-way ANOVA with Tukey’s post hoc correction). The statistical analysis performed for each data set is indicated in figure legend.

### Study approval.

All animal studies were performed according to the French and European legislation on animal care and experimentation (2010/63/EU) and approved by the local institutional ethical committee, Comité d’éthique de Genopole en expérimentation animale (CEGEA) (CEEA - 051 [Evry, France]).

### Data availability.

A Supporting Data Values file with all reported data values is available as part of the supplemental material.

## Author contributions

AG, JR, VMR, LR, PV, RL, MV, YKB, TLB, JC, JN, LJ, RF, LVW, JE, IA, and LH were involved in data generation. BB and GD were involved in rAAV production and purification. ND, XN, and GR supervised the experimental activities. AG, JR and, GR wrote the manuscript, and all authors provided review and editing of the manuscript. AG and JR contributed equally (order assigned alphabetically).

## Supplementary Material

Supplemental data

Supporting data values

## Version 1 11/28/2023

In-Press Preview

## Version 2 01/16/2024

Electronic publication
