# Supplementary material for: A functional mini-GDE transgene corrects impairment in models of glycogen storage disease type III
Source: J Clin Invest. 2024 Jan 16;134(2):e172018. doi: 10.1172/JCI172018 (PMC10786702; doi:10.1172/JCI172018)
Supplement: Supplemental data [file jci-134-172018-s201.pdf]

# Supplementary materials for

## Single AAV gene therapy with mini-Glycogen Debranching Enzyme for glycogen storage disease type III

Antoine Gardin*, Jérémy Rouillon*, Valle Montalvo-Romeral, Lucille Rossiaud, Patrice Vidal, Romain Launay, Mallaury Vie, Youssef Krimi Benchekroun, Jérémie Cosette, Bérangère Bertin, Tiziana La Bella, Guillaume Dubreuil, Justine Nozi, Louisa Jauze, Romain Fragnoud, Nathalie Daniele, Laetitia Van Wittenberghe, Jérémy Esque, Isabelle André, Xavier Nissan, Lucile Hoch, and Giuseppe Ronzitti#

* Contributed equally
# Corresponding author

**This PDF file includes:**

Fig. S1. Selection of a short promoter able to efficiently express a reporter gene in muscle
Fig. S2. High skeletal muscle transduction and strong liver de-targeting when using the rAAV-MT capsid
Fig. S3. Evaluation of the requirement for an intron in rAAV vectors encoding GDE
Fig. S4. Expression of the full-length GDE in heart and muscle of *Agl*$^{-/-}$ mice using an optimized rAAV vector
Fig. S5. Partial correction of muscle glycogen accumulation in *Agl*$^{-/-}$ mice treated with a rAAV vector encoding full-length GDE
Fig. S6. Structural organization of the human GDE protein
Fig. S7. Generation of functional truncated GDE polypeptides in the C domain
Fig. S8. Generation of functional truncated GDE polypeptides in the N-terminal domain
Fig. S9. Molecular dynamics of human GDE and ΔNter2-GDE showing conformational changes occurring along the simulation
Fig. S10. Improved productivity and quality of vectors bearing truncated ΔNter2-GDE
Fig. S11. GDE expression and glycogen reduction in skeletal muscles of the *Agl*$^{-/-}$ mouse model using rAAV vector encoding for ΔNter2-GDE
Fig. S12. Improvement of muscle histology and plasma biomarker Myom3 after injection of an rAAV vector encoding for ΔNter2-GDE in the *Agl*$^{-/-}$ mouse model
Fig. S13. Muscle correction assessed using an extended set of behavioral tests in *Agl*$^{-/-}$ mice after injection of an rAAV vector encoding for ΔNter2-GDE
Fig. S14. Absence of correction of the hepatic phenotype following injection of an rAAV-MT vector encoding for ΔNter2-GDE in the *Agl*$^{-/-}$ mouse model
Fig. S15. Correction of the muscle and heart phenotype of the *Agl*$^{-/-}$ rat model using rAAV vector encoding for ΔNter2-GDE
Fig. S16. Improvement of muscle histology and absence of cardiac toxicity in *Agl*$^{-/-}$ rats injected with an rAAV vector encoding for ΔNter2-GDE
Fig. S17. Myogenic differentiation markers and cell viability in human induced pluripotent stem cells (hiPSC)-derived muscle cells transduced with rAAV-ΔNter2-GDE
Table S1. Glycogen levels from Figure S5
Table S2. Glycogen levels from Figure 1 and Figure S7
Table S3. Glycogen levels from Figure 3 and Figure S11
Table S4. Glycogen levels from Figure 4 and Figure S15

# Supplementary Figure 1

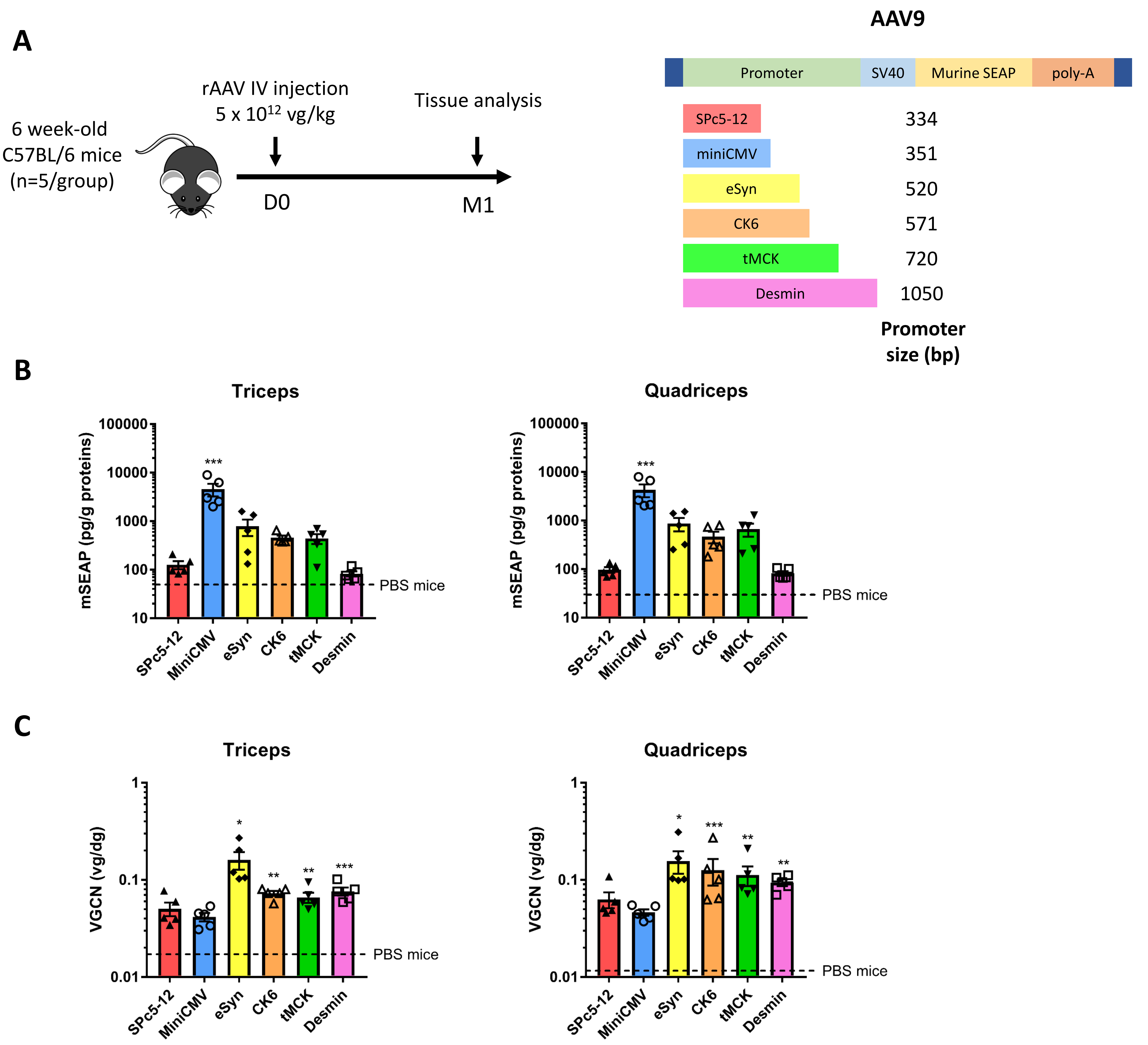


**Figure S1**. **Selection of a short promoter able to efficiently express a reporter gene in muscle.** (**A**) 6-week-old male C57BL/6 mice were injected in the tail vein with PBS or with 5 x $10^{12}$ vg/kg of rAAV9 encoding the mSEAP reporter gene under the control of various promoters including SpC512, miniCMV, eSyn, CK6, truncated muscle creatine kinase (tMCK) or Desmin (25, 26). (**B-C**) mSEAP activity quantification (**B**) and vector genome copy number (VGCN) (**C**) in triceps and quadriceps one month after vector injection. The dashed line represents the background level measured in PBS-injected mice. Statistical analyses were performed by one-way ANOVA (*p<0.05, **p<0.01 or ***p<0.001 vs. PBS-injected mice, n=5 mice per group). All data are shown as mean ±SEM. *mSEAP = mouse secreted embryonic alkaline phosphatase*

# Supplementary Figure 2

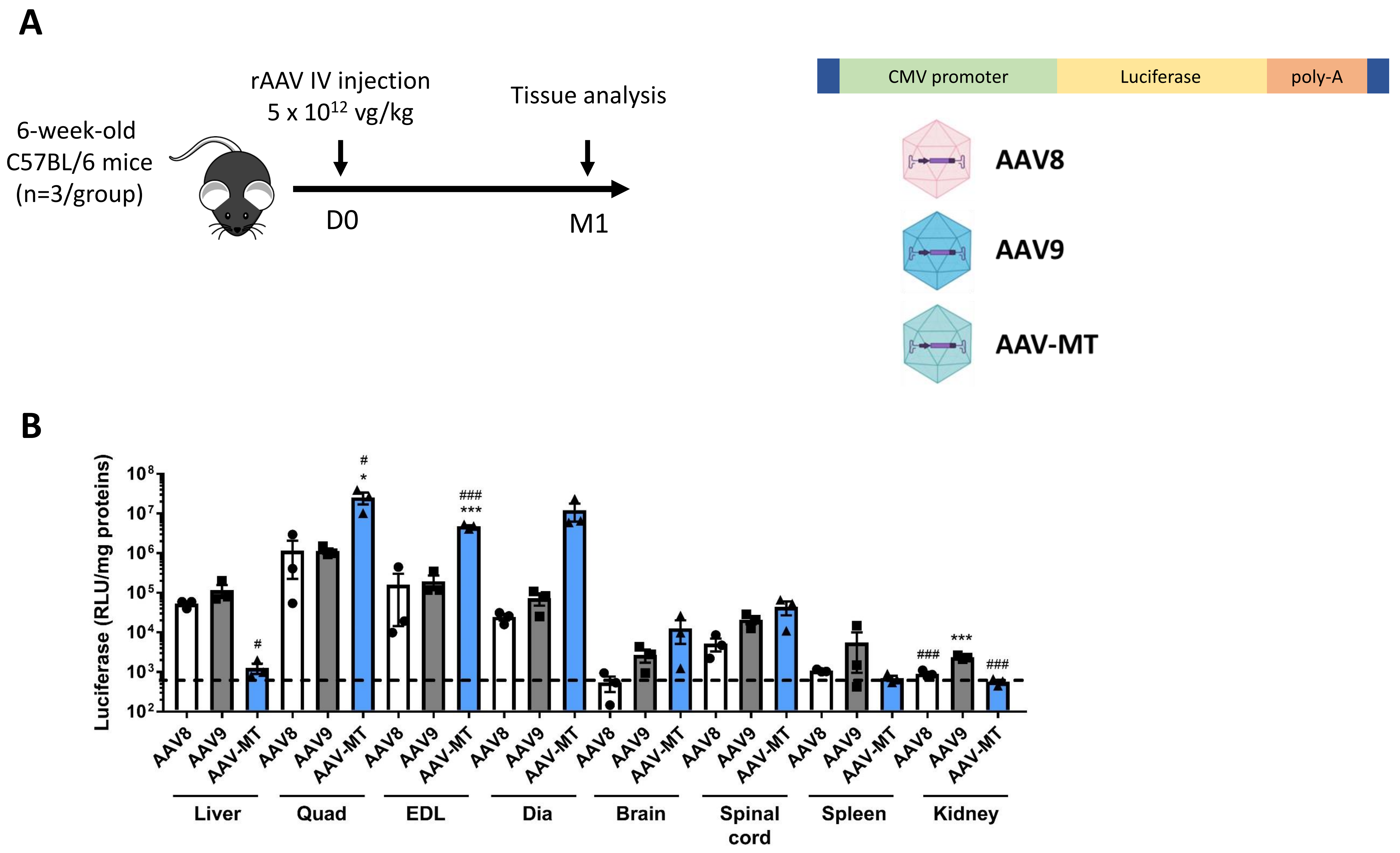


**Figure S2. High skeletal muscle transduction and strong liver de-targeting when using the rAAV-MT capsid**. (**A**) 6-week-old male C57BL/6 mice were injected in the tail vein with PBS or with 5 x $10^{12}$ vg/kg of rAAV9, rAAV8 or rAAV-MT encoding the Luciferase reporter gene under the control of the CMV promoter. (**B**) Luciferase activity quantification in different organs one month after vector injection, expressed as relative luminescence unit (RLU) normalized by total protein concentration. The dashed line represents the background level measured in PBS-injected mice. Statistical analyses were performed by one-way ANOVA (*p<0.05, **p<0.01 or ***p<0.001 vs. rAAV8-injected mice; $^{\#}$p<0.05, $^{\#\#}$p<0.01 or $^{\#\#\#}$p<0.001 vs. rAAV9-injected mice, n=3 mice per group). All data are shown as mean ±SEM. *Dia = diaphragm; EDL = extensor digitorum longus; Quad = quadriceps.*

# Supplementary Figure 3

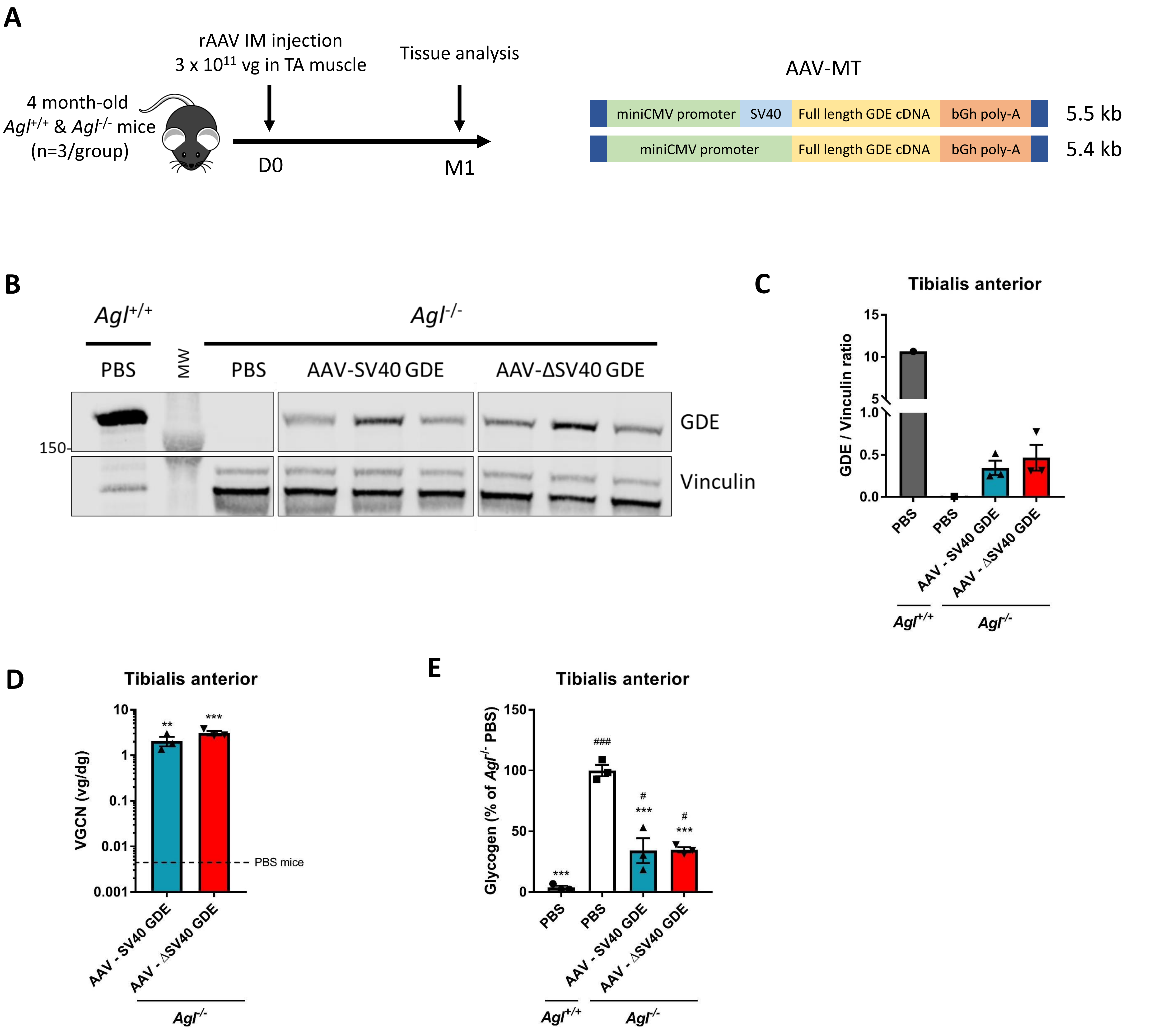


**Figure S3. Evaluation of the requirement for an intron in rAAV vectors encoding GDE.** (**A**) 4-month-old female *Agl*$^{-/-}$ mice were injected in the left tibialis anterior (TA) muscle with an rAAV-MT vector encoding the human full-length GDE with or without SV40 intron, at a dose of 3 x $10^{11}$ vg/mouse. PBS-injected *Agl*$^{-/-}$ and *Agl*$^{+/+}$ mice were used as controls. (**B**) Western Blot analysis of GDE and vinculin expression in TA muscle one month after vector injection. (**C**) Quantification of GDE expression on Western Blot in Figure S3B, expressed as the ratio of the signal of GDE and vinculin bands. (**D**) Vector genome copy number (VGCN) in the injected TA muscle. The dashed line represents the background level measured in PBS-injected mice. (**E**) Glycogen content in the injected TA muscle one month after vector injection. Statistical analyses were performed by one-way ANOVA (*p<0.05, **p<0.01 or ***p<0.001 vs. PBS-injected *Agl*$^{-/-}$ mice; $^{\#}$p<0.05, $^{\#\#}$p<0.01 or $^{\#\#\#}$p<0.001 vs. PBS-injected *Agl*$^{+/+}$, n=3 mice per group). All data are shown as mean ±SEM.

# Supplementary Figure 4

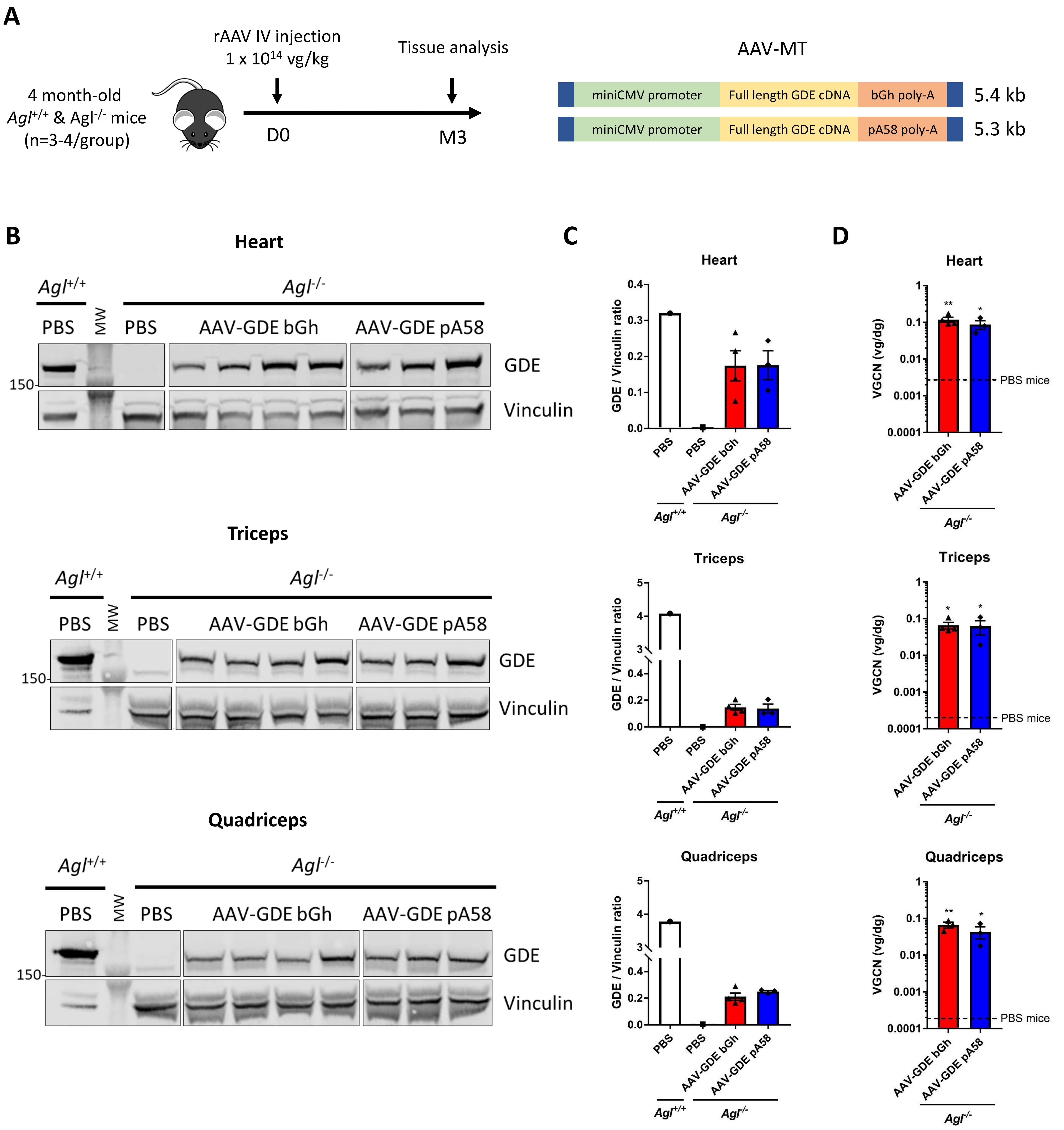


**Figure S4. Expression of the full-length GDE in heart and muscle of *Agl*$^{-/-}$ mice using an optimized rAAV vector.** (**A**) 4-month-old *Agl*$^{-/-}$ male mice were injected in the tail vein with an rAAV-MT vector encoding human full-length GDE with either the bGh or the pA58 poly-A signal, at a dose of 1 x $10^{14}$ vg/kg. PBS-injected *Agl*$^{-/-}$ and *Agl*$^{+/+}$ mice were used as controls. (**B**) Western Blot analysis of GDE and Vinculin in the heart, triceps and quadriceps 3 months after vector injection. (**C**) Quantification of GDE expression on Western Blot in Figure S4B, expressed as the ratio of the signal of GDE and vinculin bands. (**D**) Vector genome copy number (VGCN) in heart, triceps and quadriceps of injected mice. The dashed line represents the background level measured in PBS-injected mice. Statistical analyses were performed by one-way ANOVA (*p<0.05, **p<0.01 or ***p<0.001 vs. PBS-injected *Agl*$^{-/-}$ mice; $^{\#}$p<0.05, $^{\#\#}$p<0.01 or $^{\#\#\#}$p<0.001 vs. PBS-injected *Agl*$^{+/+}$ mice, n=3-4 mice per group). All data are shown as mean ±SEM.

# Supplementary Figure 5

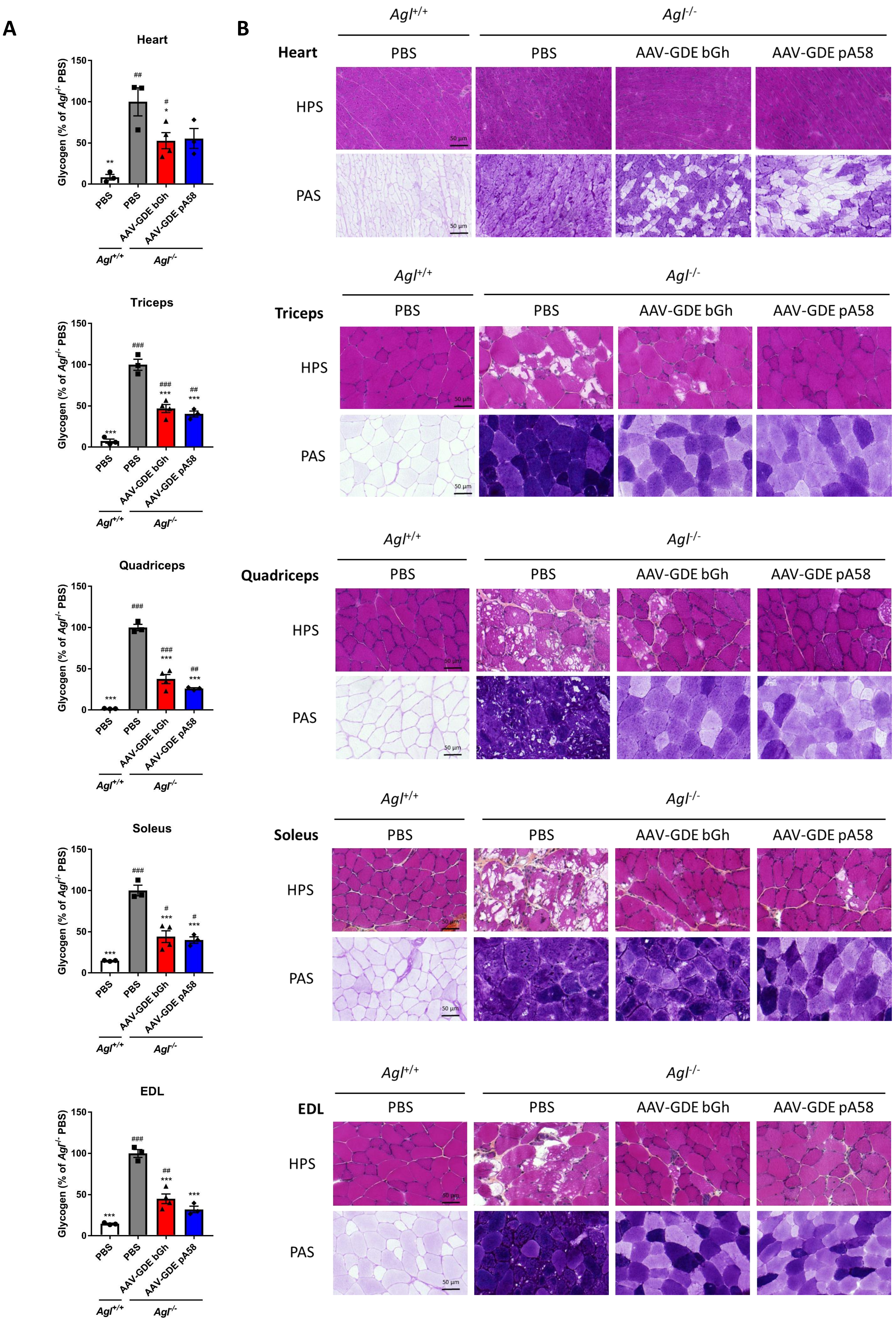


**Figure S5. Partial correction of muscle glycogen accumulation in *Agl*$^{-/-}$ mice treated with a rAAV vector encoding full-length GDE.** (**A**) Glycogen content in heart, triceps, quadriceps, soleus and EDL muscles 3 months after vector injection. (**B**) Histological analysis in heart, triceps, quadriceps, soleus and EDL using HPS and PAS staining. Representative images are shown. Statistical analyses were performed by one-way ANOVA (*p<0.05, **p<0.01 or ***p<0.001 vs. PBS-injected *Agl*$^{-/-}$ mice; $^{\#}$p<0.05, $^{\#\#}$p<0.01 or $^{\#\#\#}$p<0.001 vs. PBS-injected *Agl*$^{+/+}$ mice, n=3-4 mice per group). All data are shown as mean ±SEM. *EDL= extensor digitorum longus; HPS = hematoxylin phloxine saffron; PAS = periodic acid Schiff.*

# Supplementary Figure 6

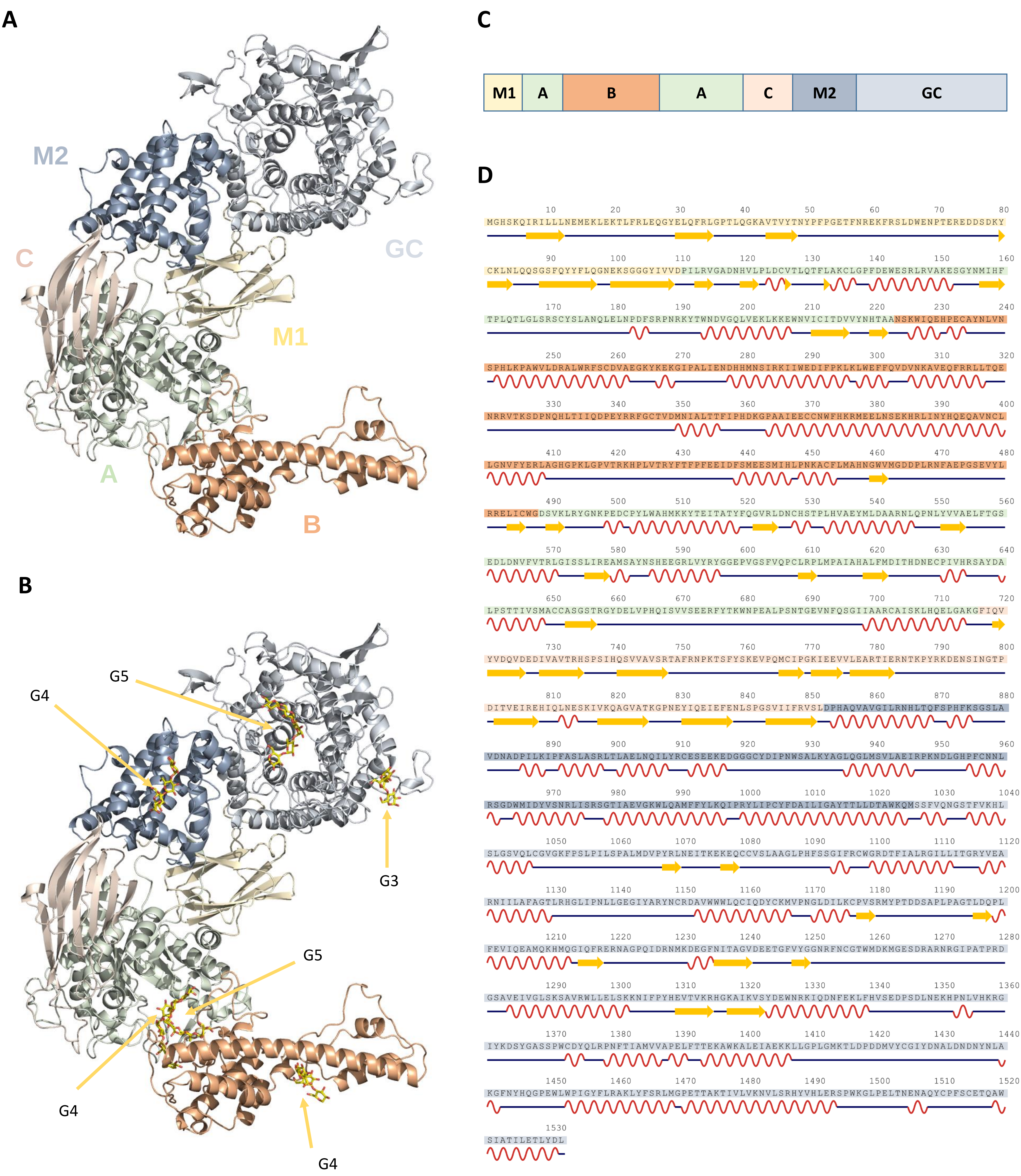


**Figure S6. Structural organization of the human GDE protein.** (**A**) Human GDE model retrieved from the AlphaFold2 database and colored according to the protein domain (**B**) Mapping onto human GDE of the sugar ligands bound in *C. glabrata* GDE crystallographic structure (PDB id: 5D0F). G3: maltotriose, G4: maltotetraose and G5: maltopentaose. (**C**) Simplified representation of the sequence of full-length GDE colored according to its domains (**D**) Amino acid sequence and secondary structures of human GDE (wave: $\alpha$-helix; arrow: $\beta$-strand), with amino acids colored according to the domain they belong to (same color code as in panel A).

# Supplementary Figure 7

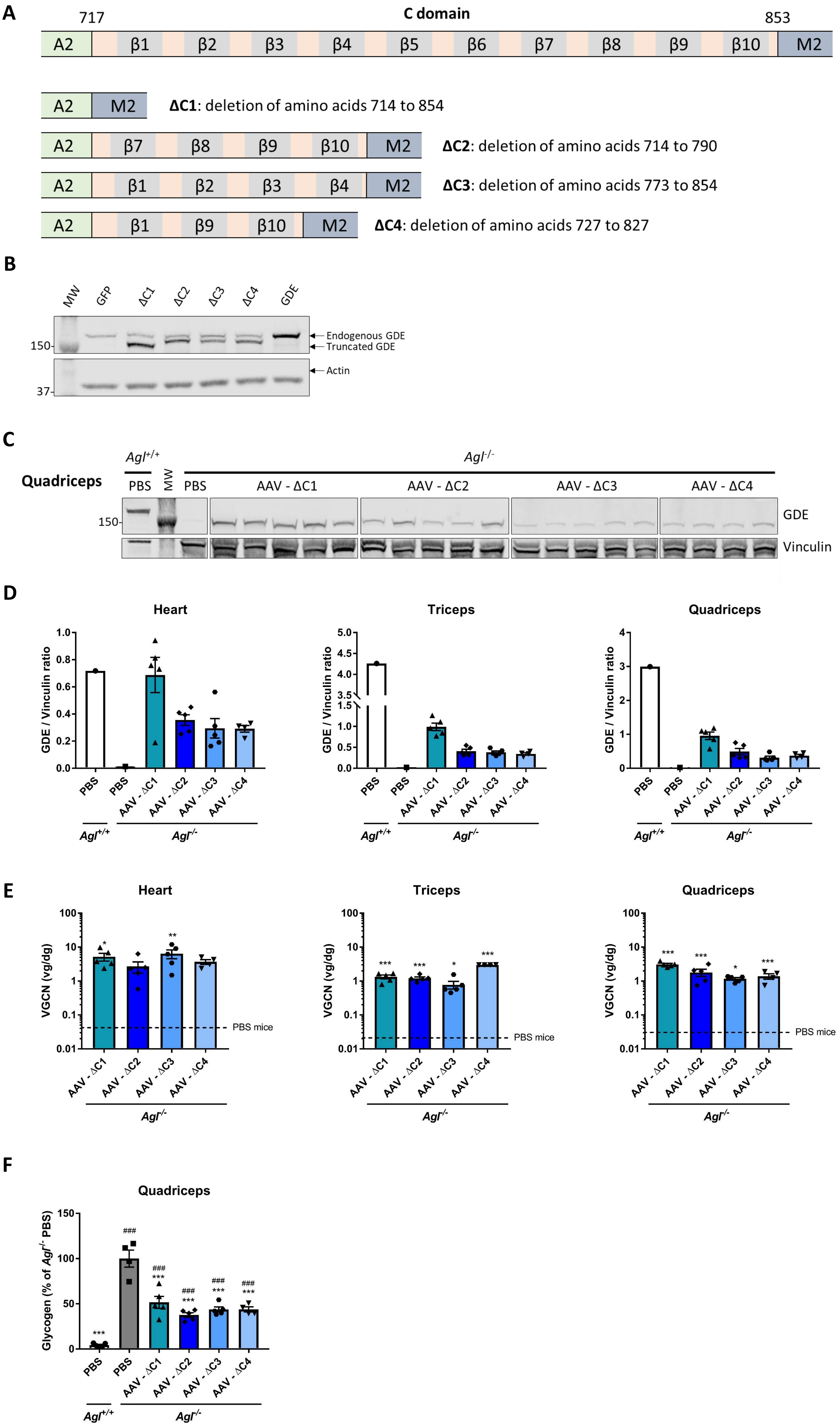

**Figure S7. Generation of functional truncated GDE polypeptides in the C domain.** (**A**) Positions of the truncation sites in the C-domain of the human GDE for mutants ΔC1 to ΔC4. (**B**) Western blot analysis of GDE and actin in HEK 293T transiently transfected with plasmids encoding green fluorescent protein (GFP), full-length GDE or ΔC1 to ΔC4 GDE deletion mutants. (**C**) Western blot analysis of GDE and Vinculin in the quadriceps three months after vector injection. (**D**) Quantification of GDE expression on Western Blot showed in Figure 1C and S7C, expressed as the ratio of the signal of GDE and vinculin bands. (**E**) Vector genome copy number (VGCN) in heart, triceps and quadriceps of injected mice. The dashed line represents the background level measured in PBS-injected mice. (**F**) Glycogen content in quadriceps 3 months after vector injection. Statistical analyses were performed by one-way ANOVA (*$p<0.05$, **$p<0.01$ or ***$p<0.001$ vs. PBS-injected *Agl*$^{-/-}$ mice; $^{\#}p<0.05$, $^{\#\#}p<0.01$ or $^{\#\#\#}p<0.001$ vs. PBS-injected *Agl*$^{+/+}$ mice, n=4-5 mice per group). All data are shown as mean ±SEM.

# Supplementary Figure 8

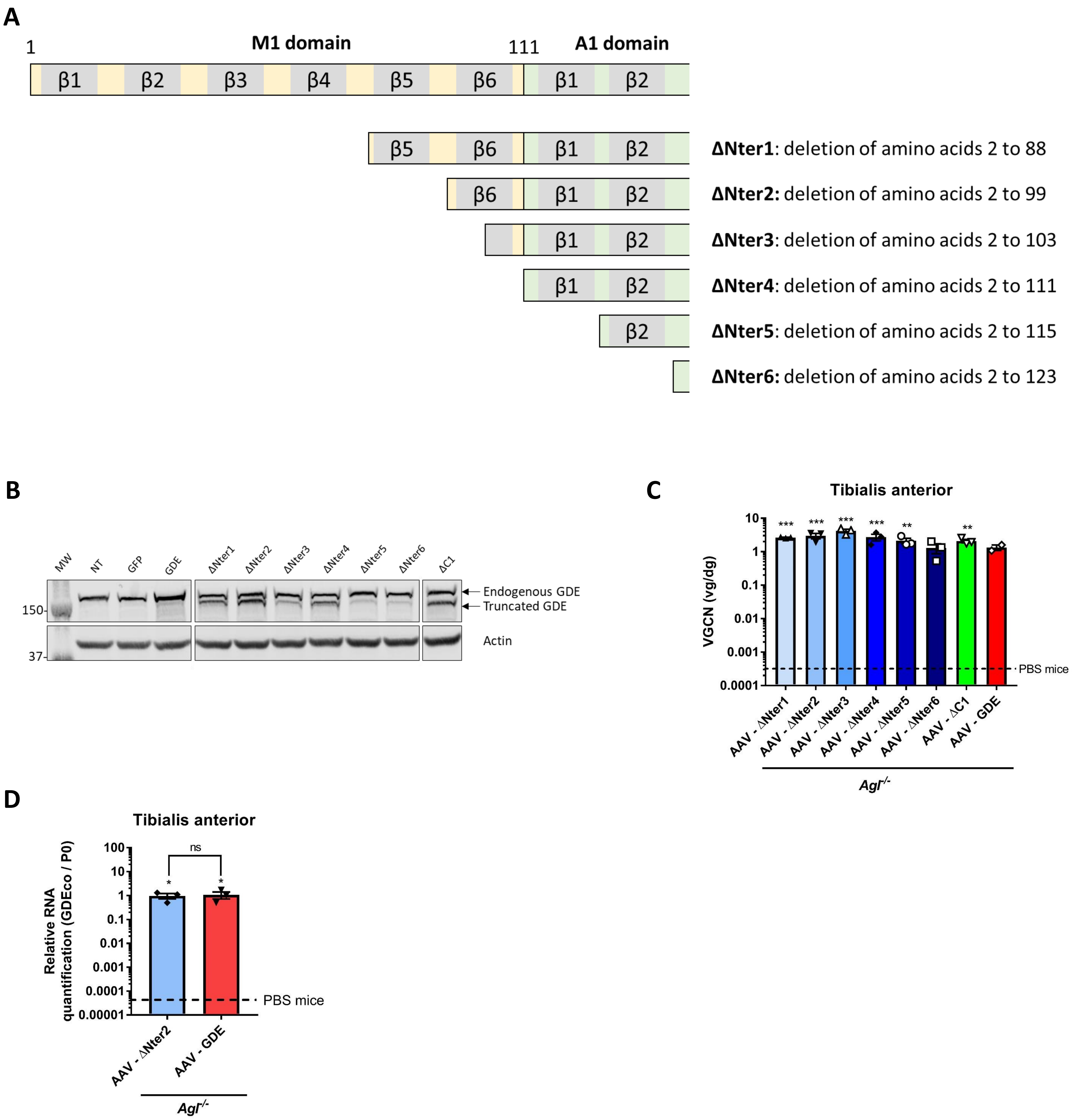


**Figure S8. Generation of functional truncated GDE polypeptides in the N-terminal domain.** (**A**) Positions of the truncation sites in the N-terminal domain of the human GDE for mutants ΔNter1 to ΔNter6. (**B**) Western blot analysis of GDE and Actin in HEK 293T transiently transfected with plasmids encoding green fluorescent protein (GFP), full-length GDE, a ΔNter1 to ΔNter6 or ΔC1 GDE mutants. NT= not transfected. (**C**) Vector genome copy number (VGCN) measured in tibialis anterior muscle of mice injected with rAAV encoding the full-length or truncated GDE. The dashed line represents the background levels measured in PBS-injected mice. (**D**) RNA expression levels of full-length or ΔNter2-truncated GDE in the injected tibialis anterior muscle. The dashed line represents the background levels measured in PBS-injected mice. Statistical analyses were performed by one-way ANOVA (*p<0.05, **p<0.01 or ***p<0.001 vs. PBS-injected $Agl^{-/-}$ mice, n=3 mice per group). All data are shown as mean ±SEM.

# Supplementary Figure 9

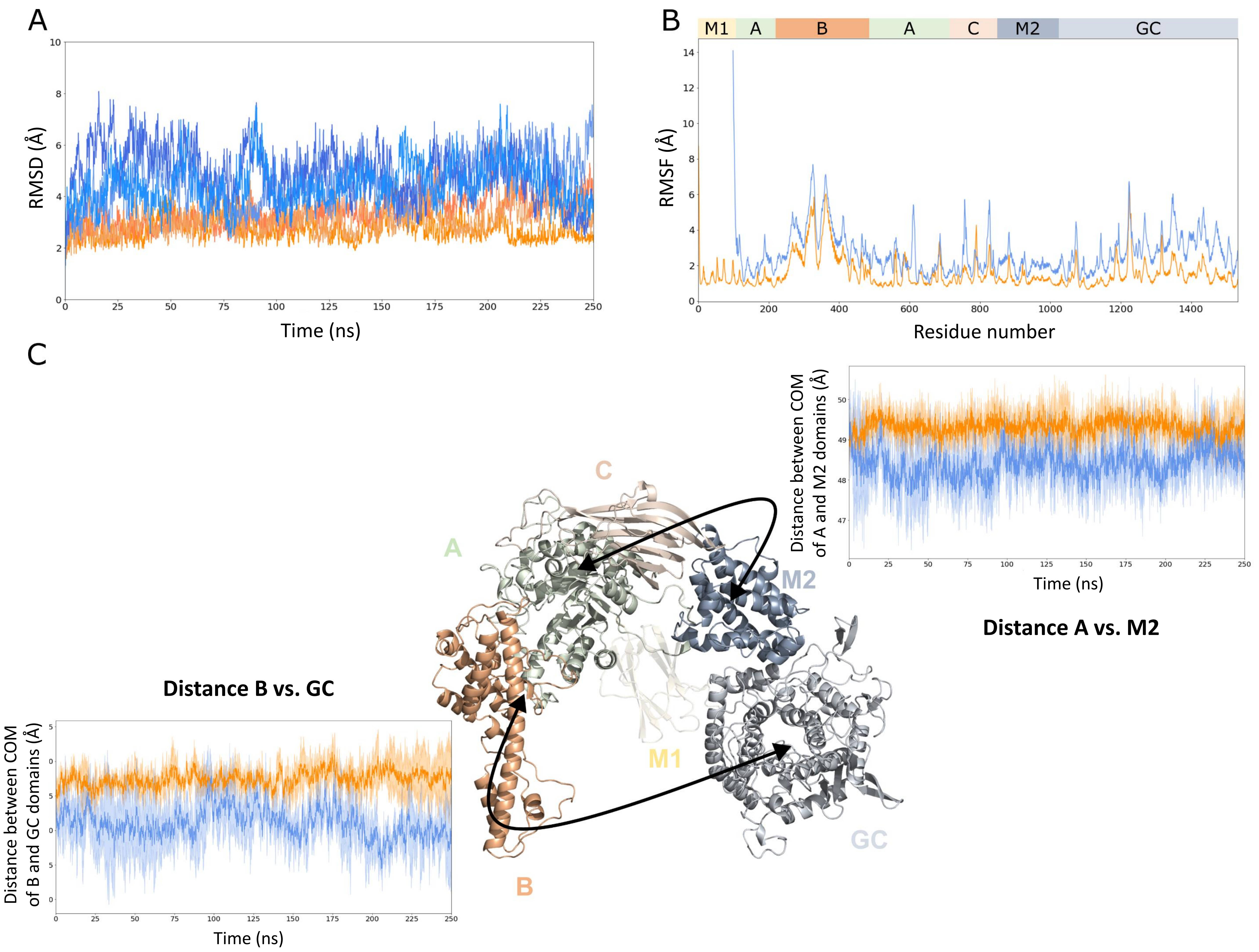


**Figure S9. Molecular dynamics of human GDE and ΔNter2-GDE showing conformational changes occurring along the simulation.** (**A**) Root mean square deviation (RMSD) computed on Cα atoms along the 250 ns of the simulation. In orange are shown the simulation replicates of full-length human GDE and in blue those of ΔNter2-GDE. (**B**) Root mean square fluctuations (RMSF) computed on amino acid residue Cα atoms along the 250 ns simulations and averaged over the triplicates of full-length human GDE (in orange) and ΔNter2-GDE (in blue). (**C**) 3D-model representation of the full-length human GDE, with in transparency the M1 domain that has been truncated in ΔNter2-GDE. The simulation of the distances between the A and M2 domains and between the B and GC domains depicted by arrows, are shown in the top right graph and the bottom left graph, respectively. Average distances were calculated on simulation triplicates at each time point between centers of mass (COM) of corresponding domains for the full-length human GDE (in orange) and ΔNter2-GDE (in blue). The standard deviation is shown as a semi-transparent area.

# Supplementary Figure 10

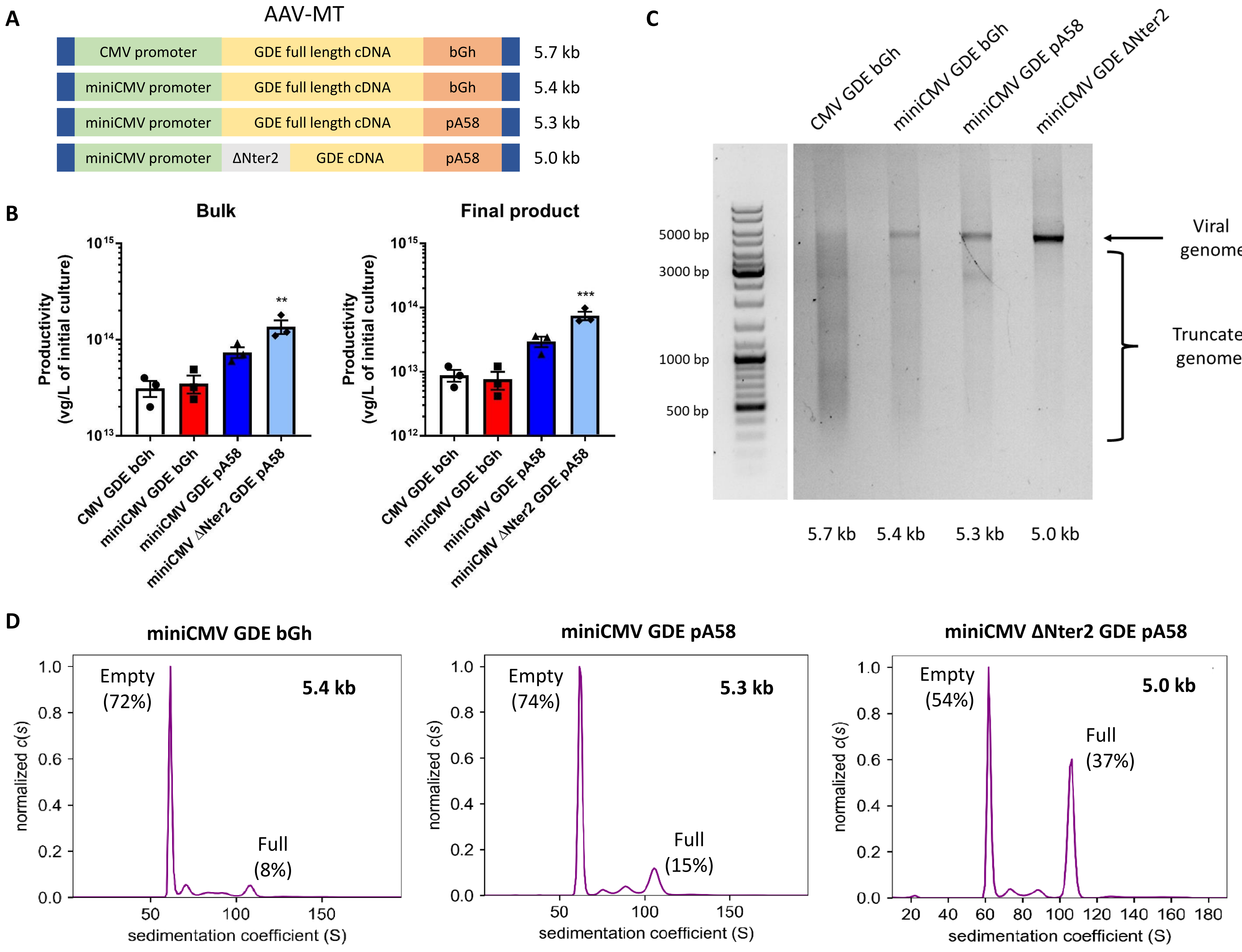


**Figure S10. Improved productivity and quality of vectors bearing truncated ΔNter2-GDE.** (**A**) Schematic representation of the four expression cassettes evaluated, with their respective size. (**B**) Small scale rAAV productions (50 mL culture) were performed in triplicate for each vector and viral titers, expressed as vector genome (vg) per liter of culture, were measured both before (bulk) and after purification (final product) for each triplicate. (**C**) rAAV vector DNA was extracted and loaded on a 1% Agarose gel to assess genome integrity. Expected genome size range from 5.0 to 5.7 kb. (**D**) Analytical ultracentrifugation analysis of the proportion of full (right peak in the magenta curve) and empty (left peak in the magenta curve) particles. Statistical analyses were performed by one-way ANOVA (*p<0.05, **p<0.01 or ***p<0.001 vs. CMV GDE bGh, experiment performed in triplicate). All data are shown as mean ±SEM.

# Supplementary Figure 11

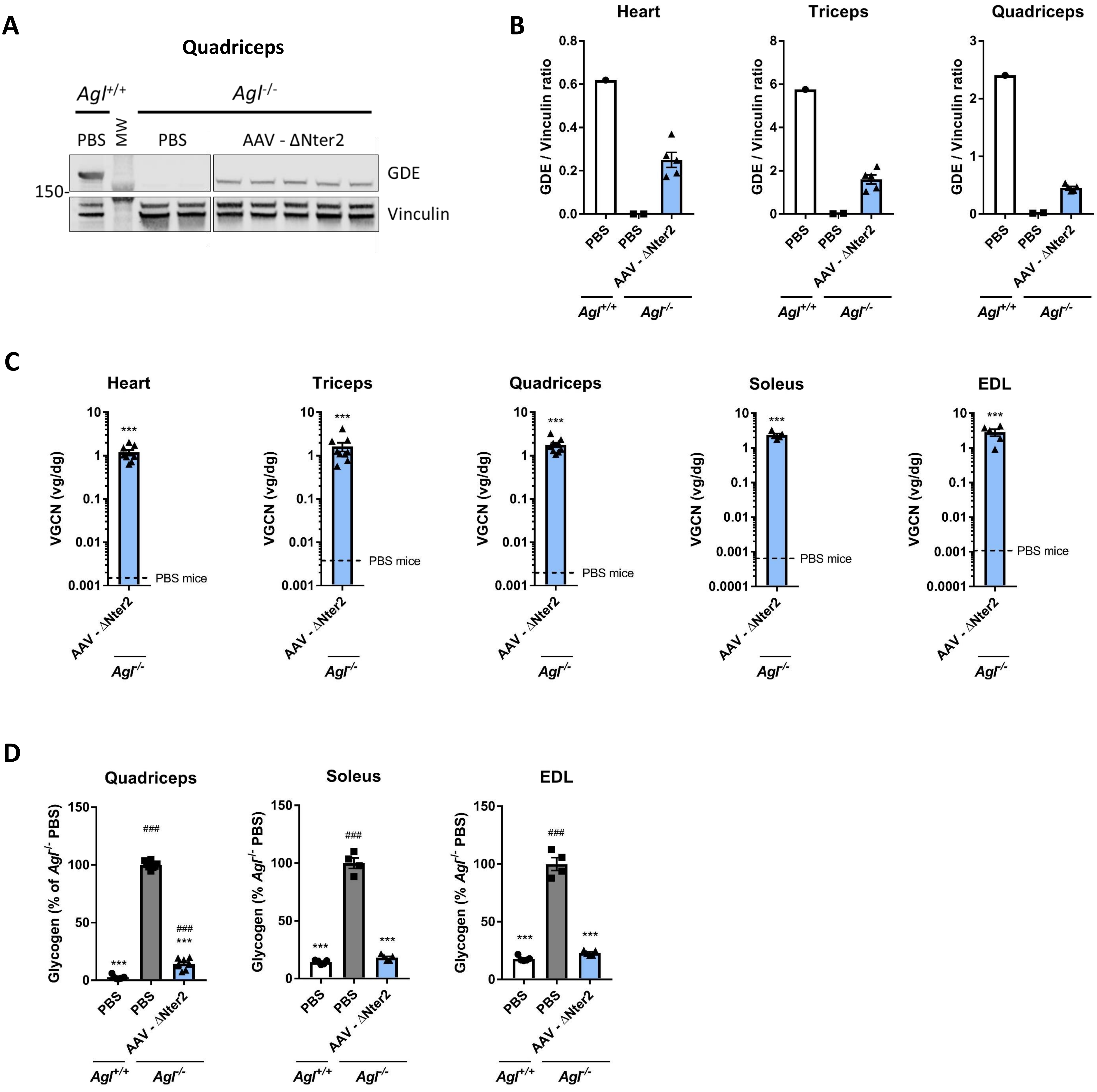


**Figure S11. GDE expression and glycogen reduction in skeletal muscles of the *Agl*$^{-/-}$ mouse model using rAAV vector encoding for ΔNter2-GDE**. (**A**) Western blot analysis of GDE and Vinculin expression in the quadriceps three months after vector injection. (**B**) Quantification of GDE expression on Western Blot showed in Figure 3B and in Figure S11A, expressed as the ratio of the signal of GDE and vinculin bands. (**C**) Vector genome copy number (VGCN) in heart, triceps, quadriceps, soleus and EDL muscles of injected mice. The dashed line represents the background level measured in PBS-injected mice. Only 4-5 mice per group (one experiment) for soleus and EDL. (**D**) Glycogen content 3 months after vector injection in quadriceps, soleus and EDL muscles. Only 4-5 mice per group (one experiment) for soleus and EDL. Statistical analyses were performed by one-way ANOVA (*p<0.05, **p<0.01 or ***p<0.001 vs. PBS-injected *Agl*$^{-/-}$ mice; $^{\#}$p<0.05, $^{\#\#}$p<0.01 or $^{\#\#\#}$p<0.001 vs. PBS-injected *Agl*$^{+/+}$ mice, n=7-9 mice per group coming from two independent experiments). All data are shown as mean ±SEM. *EDL= extensor digitorum longus.*

# Supplementary Figure 12

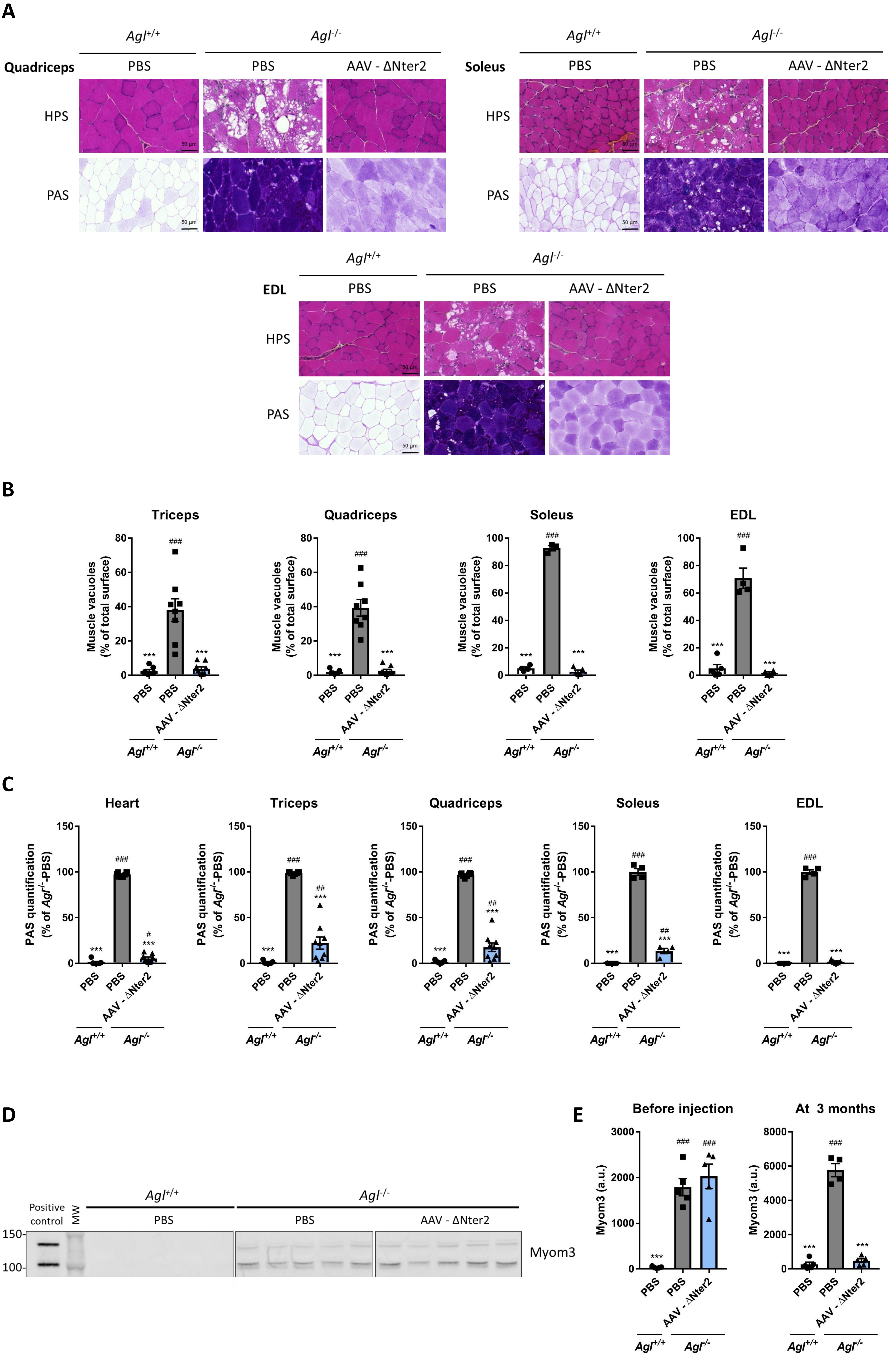


**Figure S12. Improvement of muscle histology and plasma biomarker Myom3 after injection of an rAAV vector encoding for ΔNter2-GDE in the *Agl*$^{-/-}$ mouse model** (**A**) Histological analysis of quadriceps, soleus and EDL using HPS and PAS staining. Representative images are shown. (**B**) Quantification of the architectural distortion with vacuoles seen on HPS staining of Figure 3F and Figure S12A. Only 4-5 mice per group (one experiment) for soleus and EDL. No quantification could be performed for the heart due to the absence of a defined phenotype. (**C**) Quantification of PAS staining of Figure 3E, 3F and S12A. Only 4-5 mice per group (one-

-experiment) for soleus and EDL. (**D**) Western blot analysis of Myom3 fragments in plasma of mice before injection. Plasma from mdx mouse was used as positive control. (**E**) Quantification of Myom3 expression on Western Blot showed in Figure 3G and in Figure S12D (arbitrary units). Statistical analyses were performed by one-way ANOVA (*p<0.05, **p<0.01 or ***p<0.001 vs. PBS-injected *Agl*$^{-/-}$ mice; $^{\#}$p<0.05, $^{\#\#}$p<0.01 or $^{\#\#\#}$p<0.001 vs. PBS-injected *Agl*$^{+/+}$ mice, n=7-9 mice per group coming from two independent experiments). All data are shown as mean ±SEM. *EDL= extensor digitorum longus; HPS = hematoxylin phloxine saffron; Myom3 = myomesin 3; PAS = periodic acid Schiff.*

# Supplementary Figure 13

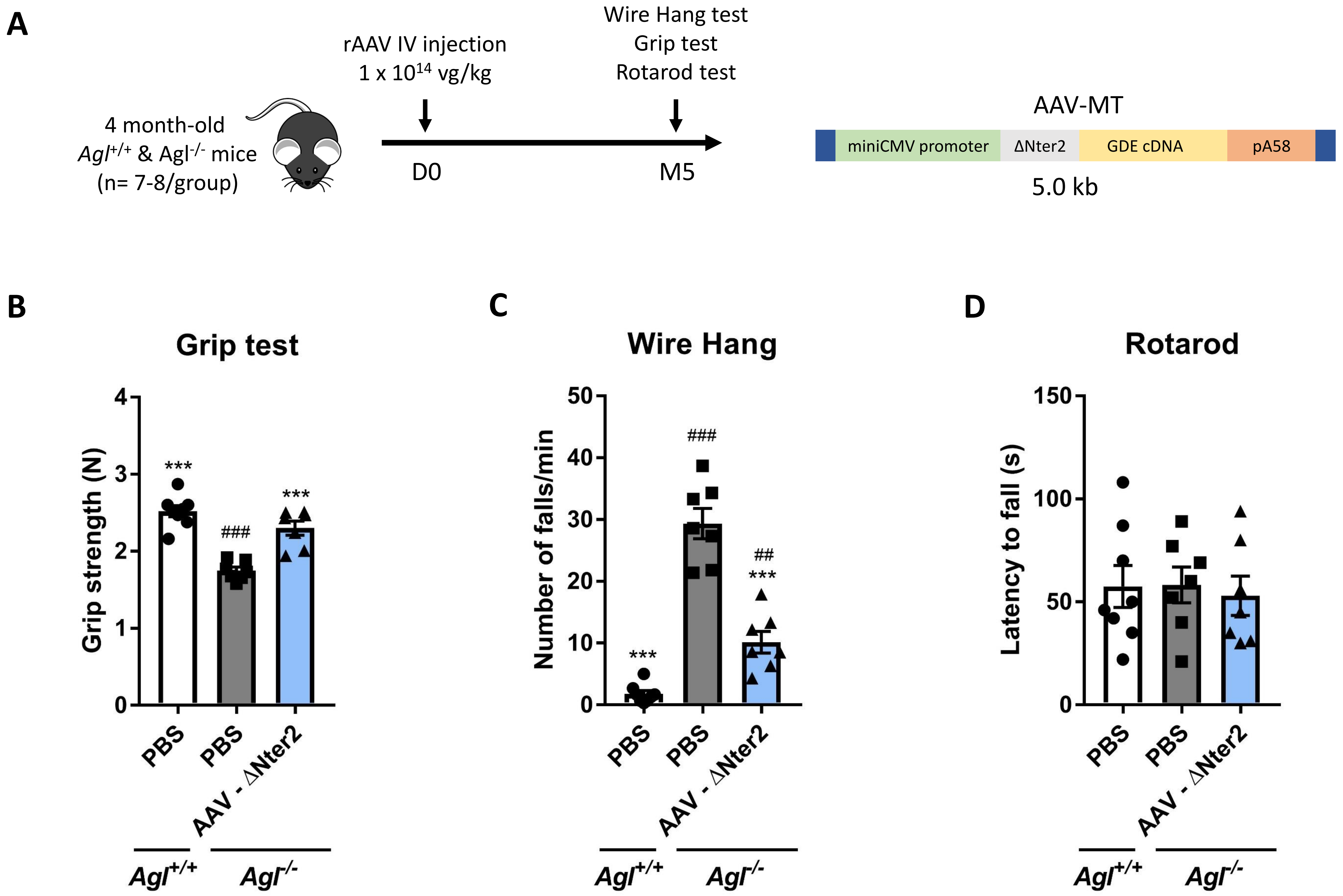


**Figure S13. Muscle correction assessed using an extended set of behavioral tests in *Agl*$^{-/-}$ mice after injection of an rAAV vector encoding for ΔNter2-GDE.** (**A**) 4-month-old male *Agl*$^{-/-}$ mice were injected in the tail vein with an rAAV-MT vector encoding ΔNter2-GDE, at the dose of 1 x $10^{14}$ vg/kg. PBS-injected *Agl*$^{+/+}$ and *Agl*$^{-/-}$ mice were used as controls. (**B**) Grip strength test, expressed as the average of three independent measures (in Newton), assessed 5 months after vector injection. (**C**) Wire-hang test, expressed as the number of falls per minute, assessed 5 months after vector injection. (**D**) Time in seconds spend on the rotarod (best performance of 3 independent experiments), assessed 5 months after vector injection. Statistical analyses were performed by one-way ANOVA (*p<0.05, **p<0.01 or ***p<0.001 vs. PBS-injected *Agl*$^{-/-}$ mice; $^{\#}$p<0.05, $^{\#\#}$p<0.01 or $^{\#\#\#}$p<0.001 vs. PBS-injected *Agl*$^{+/+}$ mice; n=7-8 mice per group). All data are shown as mean ±SEM.

# Supplementary Figure 14

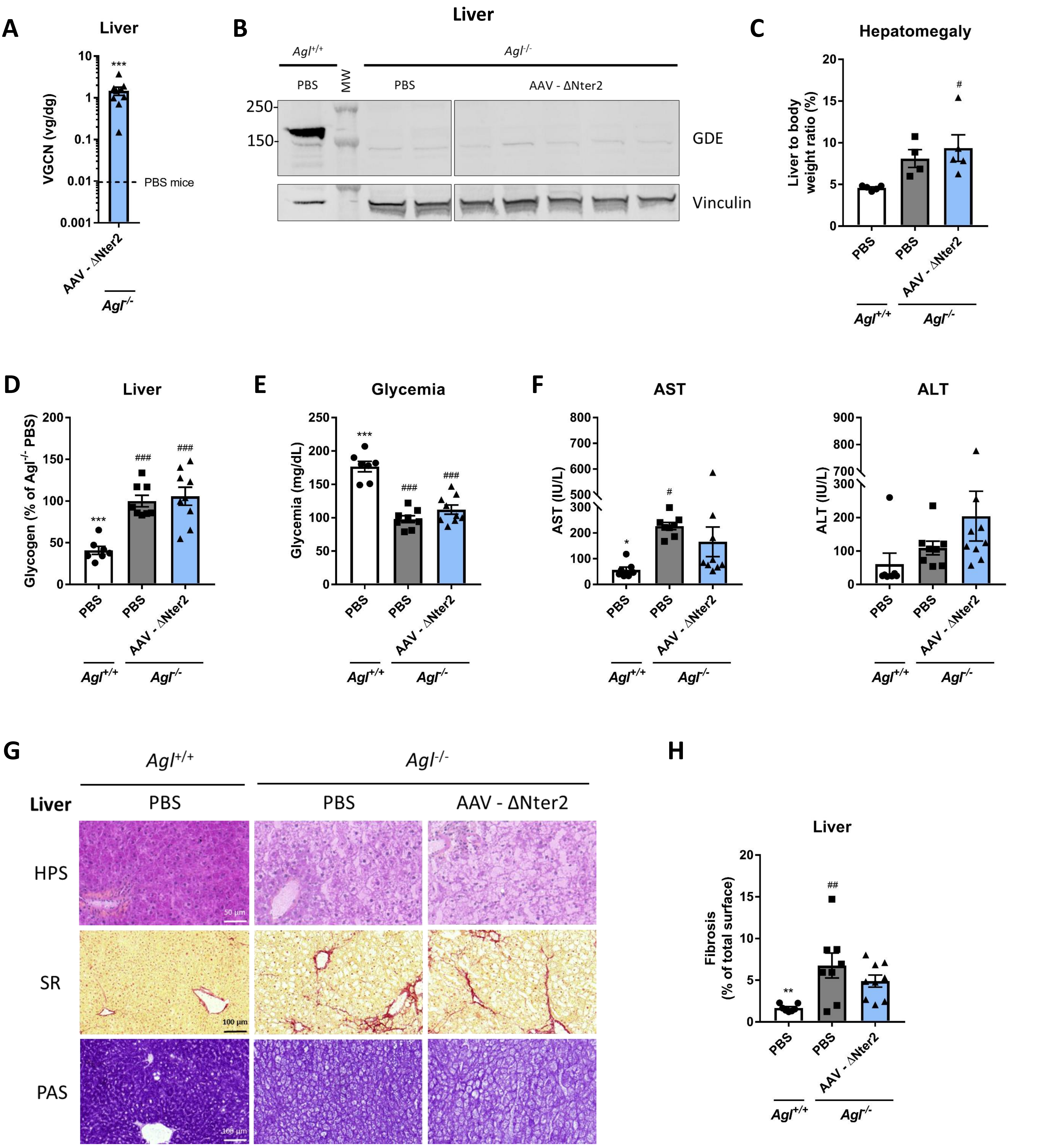


**Figure S14. Absence of correction of the hepatic phenotype following injection of an rAAV-MT vector encoding for ∆Nter2-GDE in the *Agl*$^{-/-}$ mouse model** (**A**) Vector genome copy number (VGCN) in liver three months after vector injection. The dashed line represents the background level measured in PBS-injected mice. (**B**) Western blot analysis of GDE and Vinculin in the liver three months after vector injection. (**C**) Liver to body weight ratio (reflecting hepatomegaly) 3 months after vector injection in mice injected with PBS or rAAV vector encoding for ∆Nter2-GDE. Only 4-5 mice per group (one experiment). (**D**) Glycogen content in liver 3 months after vector injection. (**E**) Glycemia measured in the fed state under anesthesia, 3 months after vector injection. (**F**) Plasma levels of aspartate aminotransferase (AST) and alanine aminotransferase (ALT) 3 months after vector injection. (**G**) Histological analysis of liver using HPS, SR and PAS staining. Representative images are shown. (**H**) Quantification of fibrosis observed on RS staining in Figure S14G. Statistical analyses were performed by one-way ANOVA (*$p<0.05$, **$p<0.01$ or ***$p<0.001$ vs. PBS-injected *Agl*$^{-/-}$ mice; $^{\#}p<0.05$, $^{\#\#}p<0.01$ or $^{\#\#\#}p<0.001$ vs. PBS-injected *Agl*$^{+/+}$ mice, n=7-9 mice per group coming from two independent experiments). All data are shown as mean ±SEM. *HPS = hematoxylin phloxine saffron; PAS = periodic acid Schiff; SR = Sirius red.*

# Supplementary Figure 15

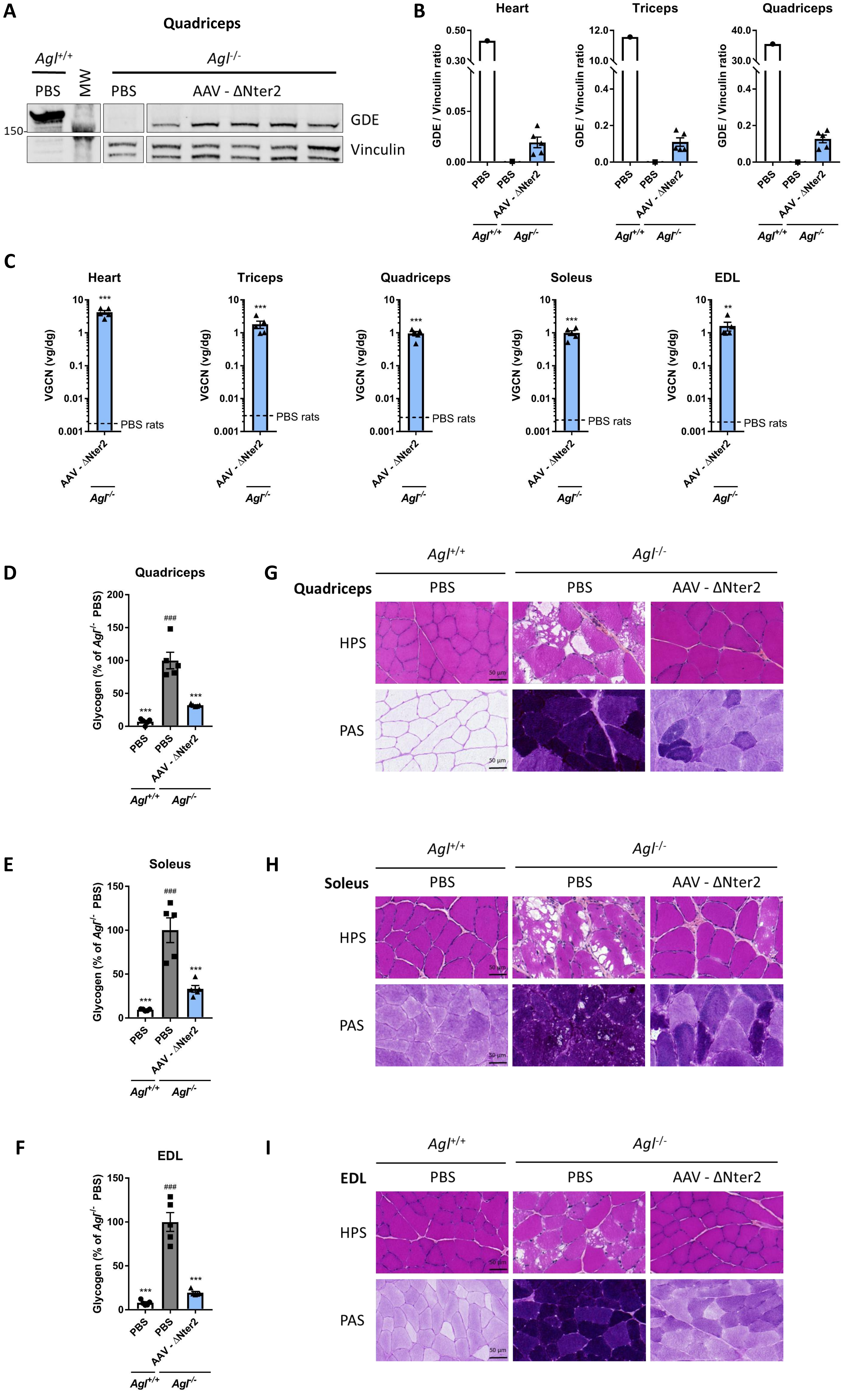

**Figure S15. Correction of the muscle and heart phenotype of the *Agl*$^{-/-}$ rat model using rAAV vector encoding for ΔNter2-GDE**. (**A**) Western blot analysis of GDE and Vinculin expression in the quadriceps three months after vector injection. (**B**) Quantification of GDE expression on Western Blot showed in Figure 4B and in Figure S15A, expressed as the ratio of the signal of GDE and vinculin bands. (**C**) Vector genome copy number (VGCN) in heart, triceps, quadriceps, soleus, and EDL muscles of injected rats. The dashed line represents the background level measured in PBS-injected rats. (**D-F**) Glycogen content in quadriceps (**D**), soleus (**E**) and EDL (**F**) muscles 3 months after vector injection. (**G-I**) Histological analysis of quadriceps (**G**), soleus (**H**) and EDL (**I**) muscles using HPS and PAS staining. Representative images are shown. Statistical analyses were performed by one-way ANOVA (*p<0.05, **p<0.01 or ***p<0.001 vs. PBS-injected *Agl*$^{-/-}$ rats; $^{\#}$p<0.05, $^{\#\#}$p<0.01 or $^{\#\#\#}$p<0.001 vs. PBS-injected *Agl*$^{+/+}$ rats, n=5 rats per group). All data are shown as mean ± SEM. *EDL= extensor digitorum longus; HPS = hematoxylin phloxine saffron; PAS = periodic acid Schiff.*

# Supplementary Figure 16

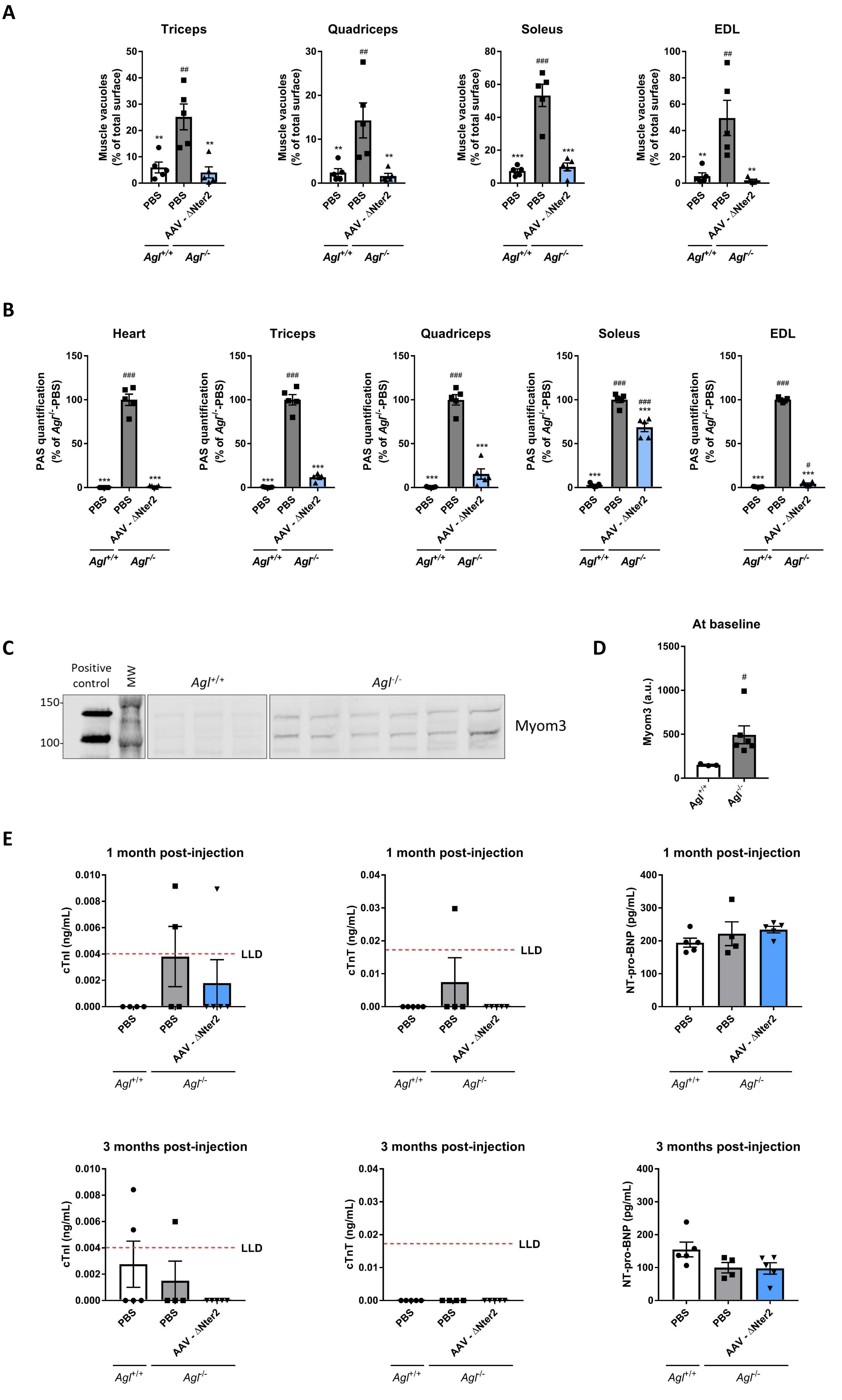

**Figure S16. Improvement of muscle histology and absence of cardiac toxicity in *Agl*$^{-/-}$ rats injected with an rAAV vector encoding for ΔNter2-GDE.** (**A**) Quantification of the architectural distortion with vacuoles seen on HPS staining of Figure 4F and Figure S15G-I. No quantification could be performed for the heart due to the absence of a defined phenotype. (**B**) Quantification of PAS staining of Figure 4E-F and S15G-I. (**C**) Western blot analysis of Myom3 fragments in plasma of 6-week-old male *Agl*$^{+/+}$ and *Agl*$^{-/-}$ rats. Plasma from mdx mouse was used as positive control (**D**) Quantification of Myom3 expression on Western Blot showed in Panel C (arbitrary units). (**E**) Quantification of cardiac troponin I (cTnI), cardiac troponin T (cTnT) and NT-pro-BNP in plasma of *Agl*$^{-/-}$ or Agl$^{+/+}$ rats injected with PBS or an rAAV vector encoding for ΔNter2-GDE at 1 or 3 months after injection. The dash line represents the lower limit of detection (LLD). Statistical analyses were performed by one-way ANOVA in panels A-B-E (*p<0.05, **p<0.01 or ***p<0.001 vs. PBS-injected *Agl*$^{-/-}$ rats; $^{\#}$p<0.05, $^{\#\#}$p<0.01 or $^{\#\#\#}$p<0.001 vs. PBS-injected *Agl*$^{+/+}$ rats; n=4-5 rats per group) and by Welch T-test in panel D ($^{\#}$p<0.05, $^{\#\#}$p<0.01 or $^{\#\#\#}$p<0.001, n=3 Agl$^{+/+}$ rats and 6 Agl$^{-/-}$ rats). All data are shown as mean ±SEM. *Myom3 = myomesin 3; HPS = hematoxylin phloxine saffron; PAS = periodic acid Schiff;*

# Supplementary Figure 17

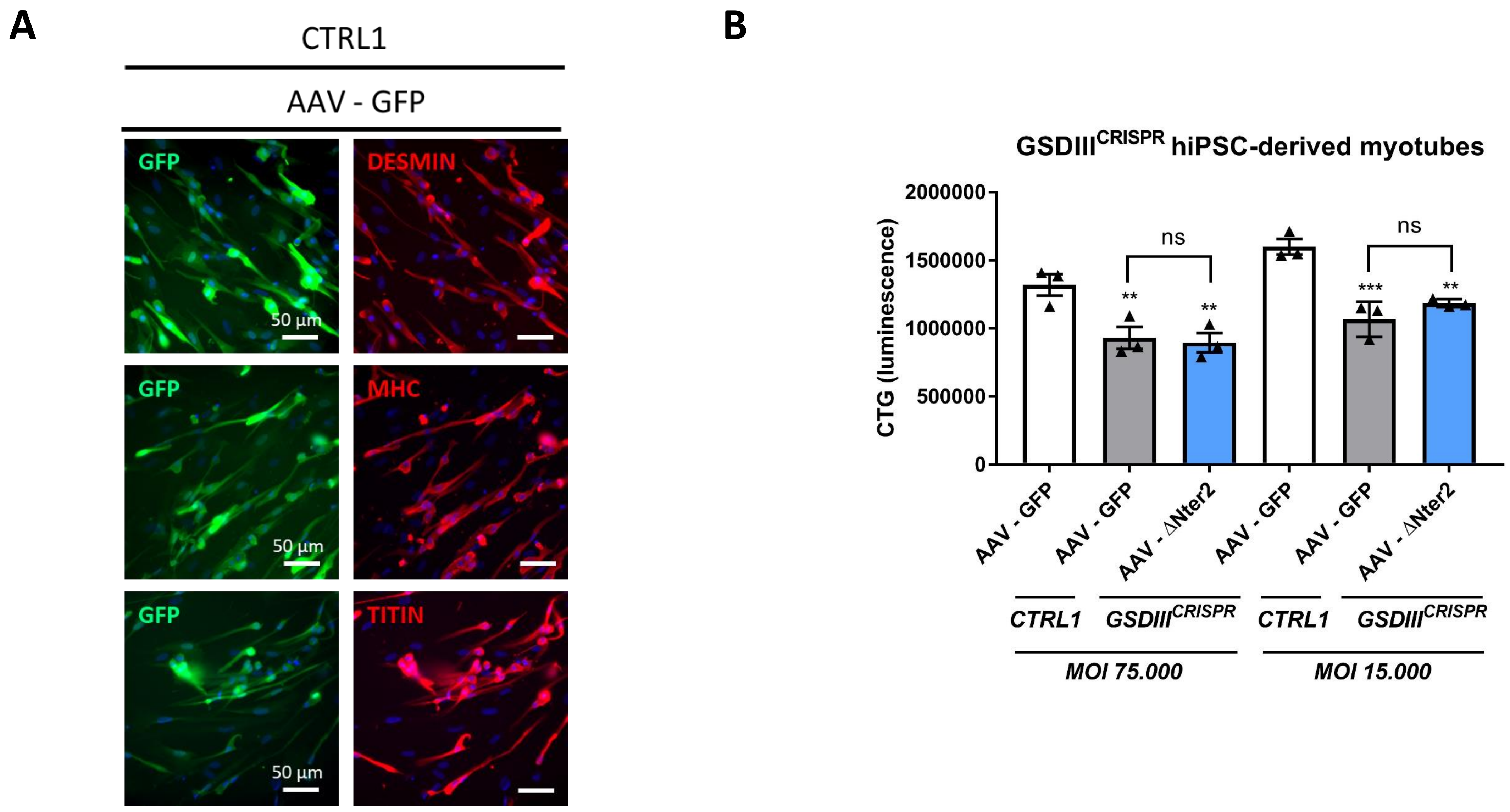


**Figure S17. Myogenic differentiation markers and cell viability in human induced pluripotent stem cells (hiPSC)-derived muscle cells transduced with rAAV-ΔNter2-GDE.** (**A**) Expression of specific myogenic markers (desmin, myosin heavy chain (MHC) and titin) and green fluorescent protein (GFP) by immuno-fluorescence analysis, after rAAV transduction at a multiplicity of infection (MOI) of 75 000, in CTRL1 hiPSC-derived skMb. Representative images are shown (**B**) Cell viability assessed by evaluating ATP levels in GSDIII$^{CRISPR}$ hiPSC-derived skMt and isogenic controls (CTRL1) transduced as described in **Figure 5**. Statistical analyses were performed by one-way ANOVA (*p<0.05, **p<0.01 or ***p<0.001 vs. AAV-GFP-transduced CTRL1 cells). Experiments performed in triplicate. All data are shown as mean ±SEM.

**Table S1. Glycogen levels from Figure S5**

| | | Glycogen (mmol glucose/g proteins) | | | | |
|---|---|---|---|---|---|---|
| **Genotype** | **Treatment (n)** | **Heart** | **Triceps** | **Quadriceps** | **Soleus** | **EDL** |
| *Agl*$^{+/+}$ | PBS (n=3) | 0.04 ± 0.02** | 0.15 ± 0.05*** | 0.06 ± 0.01*** | 0.38 ± 0.01*** | 0.33 ± 0.02*** |
| *Agl*$^{-/-}$ | PBS (n=3) | 0.45 ± 0.08$^{\#\#}$ | 2.11 ± 0.14$^{\#\#\#}$ | 3.49 ± 0.14$^{\#\#\#}$ | 2.55 ± 0.16$^{\#\#\#}$ | 2.27 ± 0.11$^{\#\#\#}$ |
| | AAV-GDE bGh (n=4) | 0.24 ± 0.04*$^{,\#}$ | 0.99 ± 0.1***$^{,\#\#\#}$ | 1.32 ± 0.19***$^{,\#\#\#}$ | 1.12 ± 0.18***$^{,\#}$ | 1.02 ± 0.13***$^{,\#\#}$ |
| | AAV-GDE pA58 (n=3) | 0.25 ± 0.06 | 0.85 ± 0.08***$^{,\#\#}$ | 0.91 ± 0.04***$^{,\#\#}$ | 1.02 ± 0.1***$^{,\#}$ | 0.72 ± 0.09*** |

Values are shown as Mean ± SEM. Statistical analyses were performed by one-way ANOVA (*p<0.05, **p<0.01 or ***p<0.001 vs. PBS-injected *Agl*$^{-/-}$ mice; $^{\#}$p<0.05, $^{\#\#}$p<0.01 or $^{\#\#\#}$p<0.001 vs. PBS-injected *Agl*$^{+/+}$ mice, n=3-4 mice per group). *EDL= extensor digitorum longus*.

**Table S2. Glycogen levels from Figure 1 and Figure S7**

| | | Glycogen (mmol glucose/g proteins) | | |
|---|---|---|---|---|
| Genotype | Treatment (n) | Heart | Triceps | Quadriceps |
| *Agl*$^{+/+}$ | PBS (n=5) | 0.002 ± 0.002*** | 0.12 ± 0.02*** | 0.11 ± 0.02*** |
| *Agl*$^{-/-}$ | PBS (n=4) | 0.85 ± 0.14$^{\#\#\#}$ | 2.71 ± 0.26$^{\#\#\#}$ | 2.57 ± 0.24$^{\#\#\#}$ |
| | AAV-ΔC1 (n=5) | 0.66 ± 0.04$^{\#\#\#}$ | 0.71 ± 0.07***$^{,\#}$ | 1.33 ± 0.17***$^{,\#\#\#}$ |
| | AAV-ΔC2 (n=5) | 0.75 ± 0.1$^{\#\#\#}$ | 0.99 ± 0.11***$^{,\#\#\#}$ | 0.96 ± 0.07***$^{,\#\#\#}$ |
| | AAV-ΔC3 (n=5) | 0.98 ± 0.14$^{\#\#\#}$ | 1.52 ± 0.17***$^{,\#\#\#}$ | 1.12 ± 0.07***$^{,\#\#\#}$ |
| | AAV-ΔC4 (n=4) | 0.76 ± 0.04$^{\#\#\#}$ | 0.98 ± 0.05***$^{,\#\#\#}$ | 1.13 ± 0.07***$^{,\#\#\#}$ |

Values are shown as Mean ± SEM. Statistical analyses were performed by one-way ANOVA (*p<0.05, **p<0.01 or ***p<0.001 vs. PBS-injected *Agl*$^{-/-}$ mice; $^{\#}$p<0.05, $^{\#\#}$p<0.01 or $^{\#\#\#}$p<0.001 vs. PBS-injected *Agl*$^{+/+}$ mice, n=4-5 mice per group).

**Table S3. Glycogen levels from Figure 3 and Figure S11**

| | | Glycogen (mmol glucose/g proteins) | | | | |
|---|---|---|---|---|---|---|
| **Genotype** | **Treatment (n)** | **Heart** | **Triceps** | **Quadriceps** | **Soleus#** | **EDL#** |
| *Agl*$^{+/+}$ | PBS (n=7) | 0.07 ± 0.02*** | 0.09 ± 0.02*** | 0.09 ± 0.02*** | 0.89 ± 0.05*** | 0.71 ± 0.04*** |
| *Agl*$^{-/-}$ | PBS (n=8) | 1.06 ± 0.09### | 3.18 ± 0.06### | 3.35 ± 0.04### | 6.19 ± 0.28### | 3.97 ± 0.22### |
| | AAV-ΔNter2 (n=9) | 0.25 ± 0.02*** | 0.52 ± 0.05***,### | 0.48 ± 0.05***,### | 1.13 ± 0.07*** | 0.91 ± 0.04*** |

Values are shown as Mean ± SEM. Statistical analyses were performed by one-way ANOVA (*p<0.05, **p<0.01 or ***p<0.001 vs. PBS-injected *Agl*$^{-/-}$ mice; #p<0.05, ##p<0.01 or ###p<0.001 vs. PBS-injected *Agl*$^{+/+}$ mice, n=7-9 mice per group). *EDL= extensor digitorum longus.*

#Only four to five mice per group (one experiment) had sampling of soleus and EDL.

**Table S4. Glycogen levels from Figure 4 and Figure S15**

| | | Glycogen (mmol glucose/g proteins) | | | | |
|---|---|---|---|---|---|---|
| **Genotype** | **Treatment (n)** | **Heart** | **Triceps** | **Quadriceps** | **Soleus** | **EDL** |
| *Agl*$^{+/+}$ | PBS (n=5) | 0.15 ± 0.05*** | 0.1 ± 0.01*** | 0.1 ± 0.03*** | 0.46 ± 0.02*** | 0.2 ± 0.03*** |
| *Agl*$^{-/-}$ | PBS (n=5) | 1.25 ± 0.21$^{###}$ | 1.99 ± 0.33$^{###}$ | 1.42 ± 0.18$^{###}$ | 4.87 ± 0.69$^{###}$ | 2.52 ± 0.27$^{###}$ |
| | AAV-ΔNter2 (n=5) | 0.65 ± 0.07$^{*,\#}$ | 0.41 ± 0.01*** | 0.46 ± 0.01*** | 1.62 ± 0.19*** | 0.49 ± 0.04*** |

Values are shown as Mean ± SEM. Statistical analyses were performed by one-way ANOVA (*p<0.05, **p<0.01 or ***p<0.001 vs. PBS-injected *Agl*$^{-/-}$ rats; $^{\#}$p<0.05, $^{\#\#}$p<0.01 or $^{\#\#\#}$p<0.001 vs. PBS-injected *Agl*$^{+/+}$ rats, n=5 rats per group). *EDL= extensor digitorum longus.*
